# Supplementary figures and images for: An automated, high-throughput method for standardizing image color profiles to improve image-based plant phenotyping
Source: PeerJ. 2018 Oct 4;6:e5727. doi: 10.7717/peerj.5727 (PMC6174877; doi:10.7717/peerj.5727)

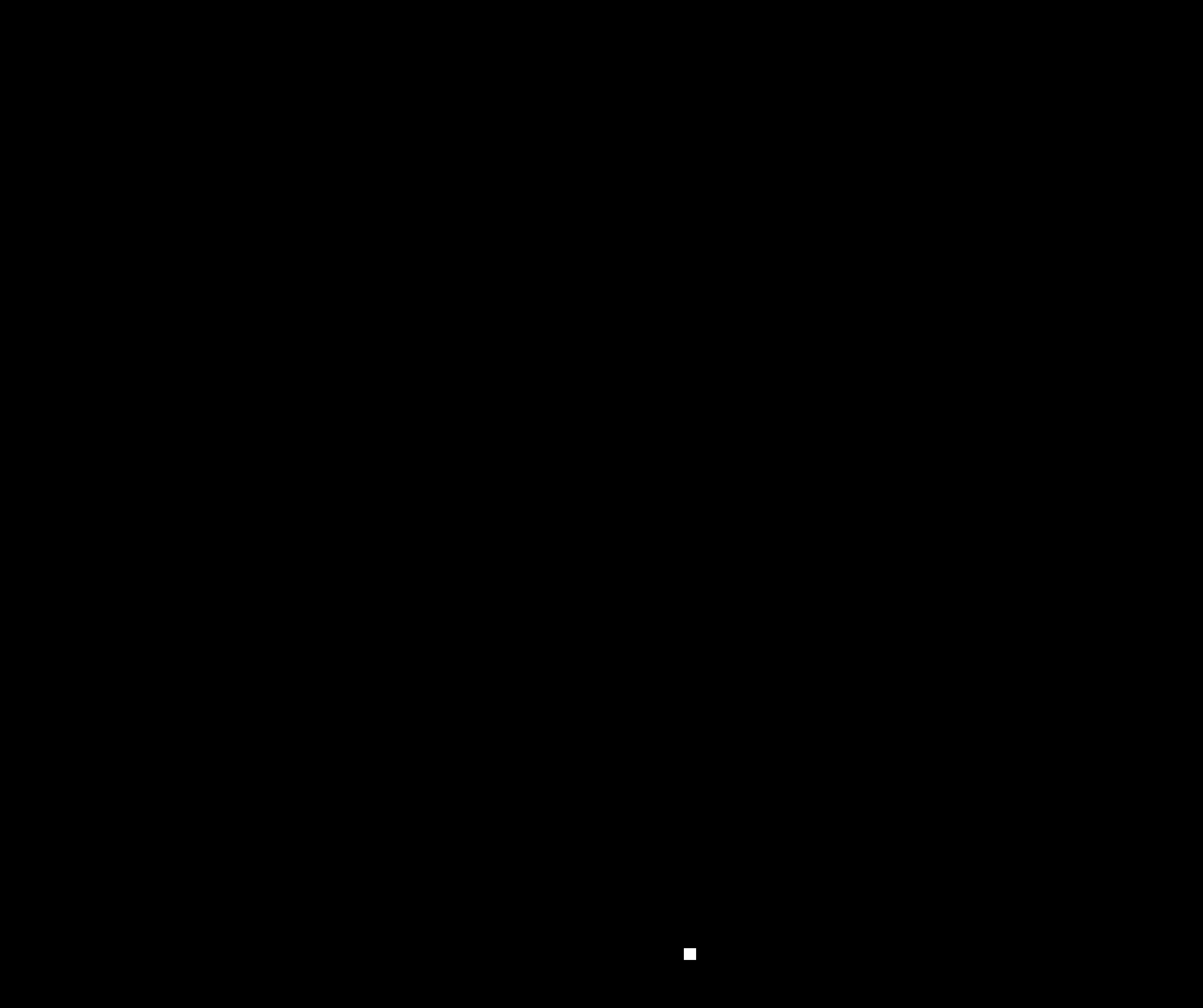

Supplement: Supplemental Information 1 — The supplemental zip file contains 3 folders: data, scripts, and license. The scripts enable denovo analysis of the data contained in the data folder, which was used to generate the figures in the manuscript. The license is GPL version2. [file peerj-06-5727-s001.zip › analysis/data/plant/card_masks/10_mask.png]

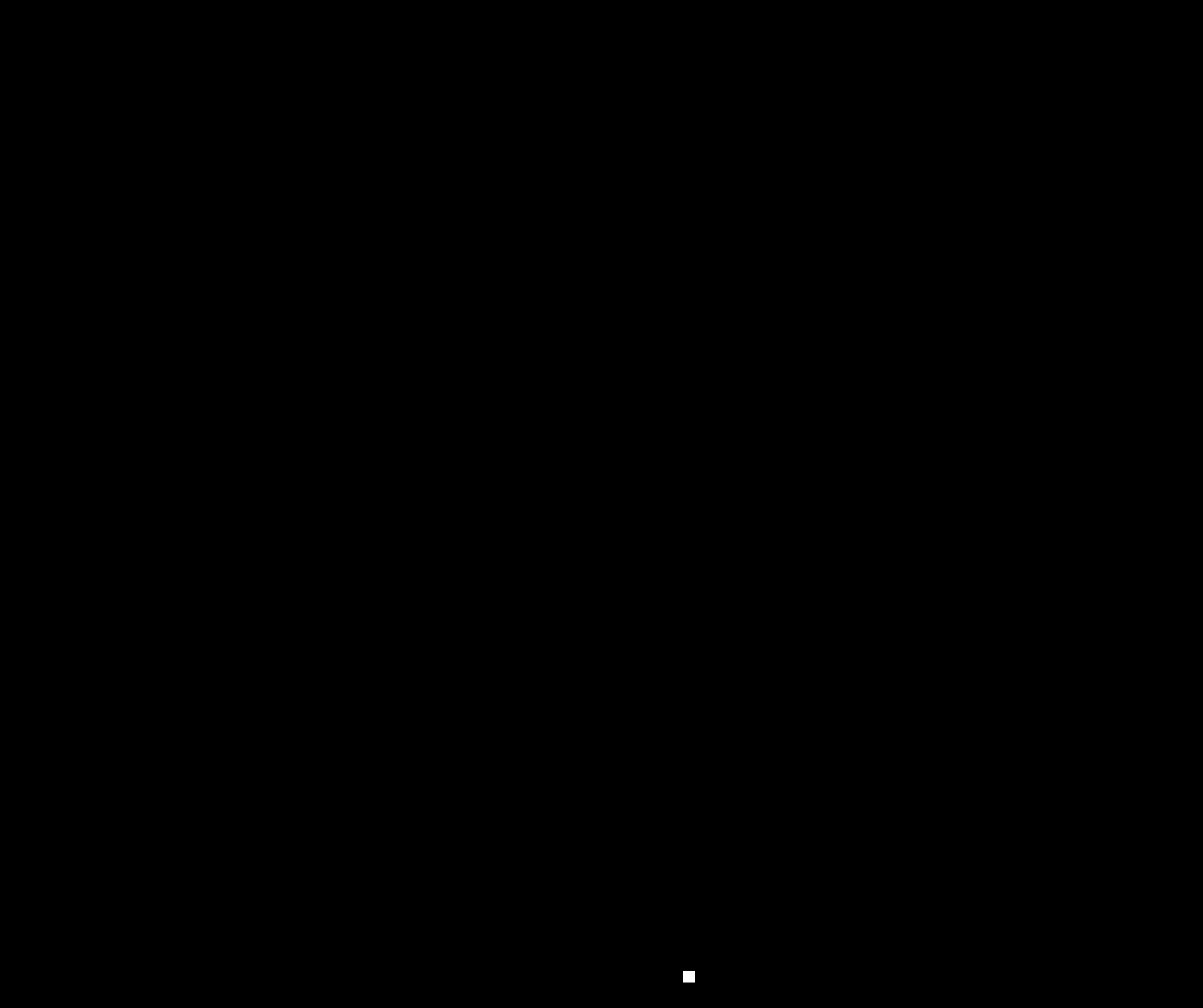

Supplement: Supplemental Information 1 — The supplemental zip file contains 3 folders: data, scripts, and license. The scripts enable denovo analysis of the data contained in the data folder, which was used to generate the figures in the manuscript. The license is GPL version2. [file peerj-06-5727-s001.zip › analysis/data/plant/card_masks/11_mask.png]

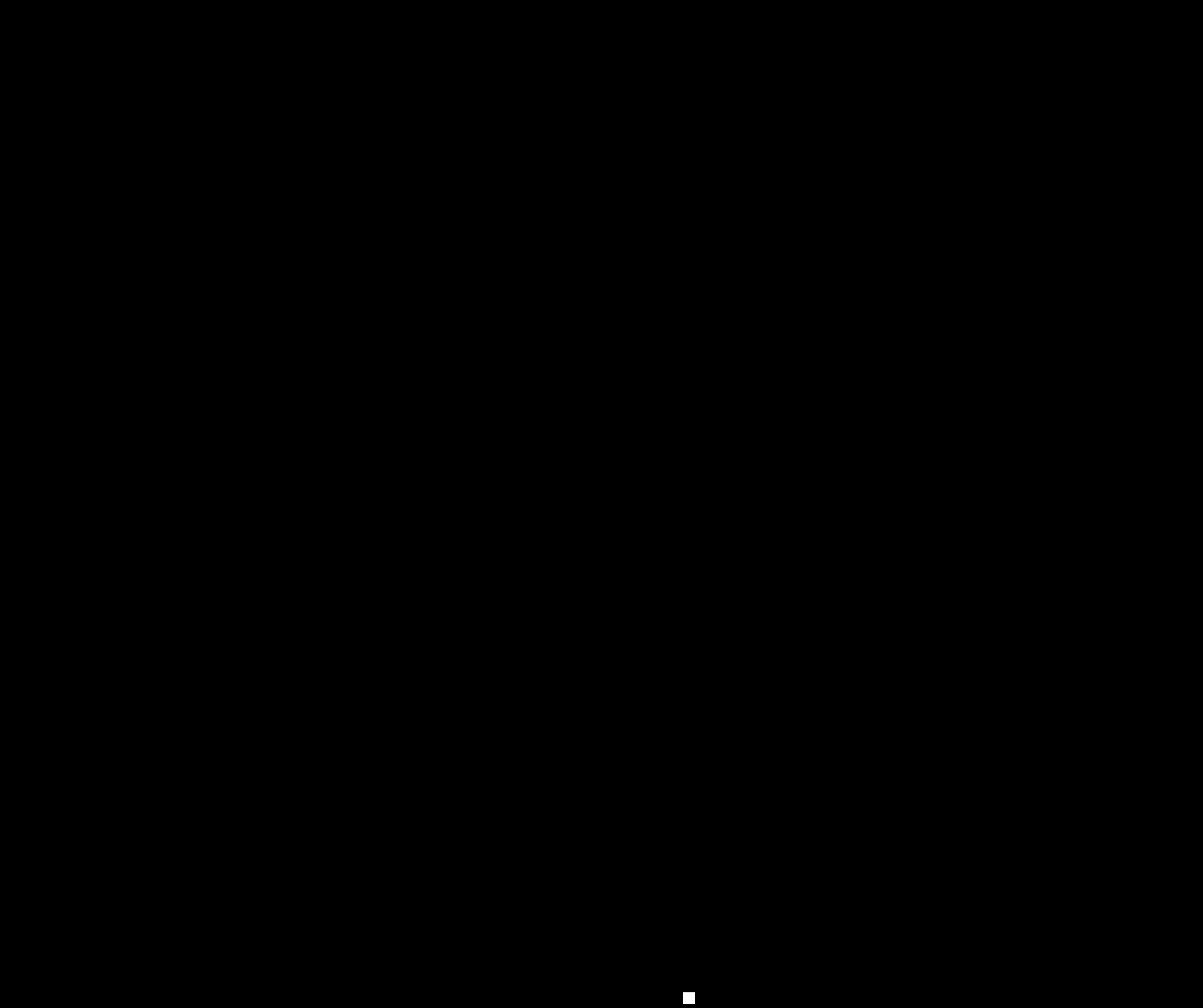

Supplement: Supplemental Information 1 — The supplemental zip file contains 3 folders: data, scripts, and license. The scripts enable denovo analysis of the data contained in the data folder, which was used to generate the figures in the manuscript. The license is GPL version2. [file peerj-06-5727-s001.zip › analysis/data/plant/card_masks/12_mask.png]

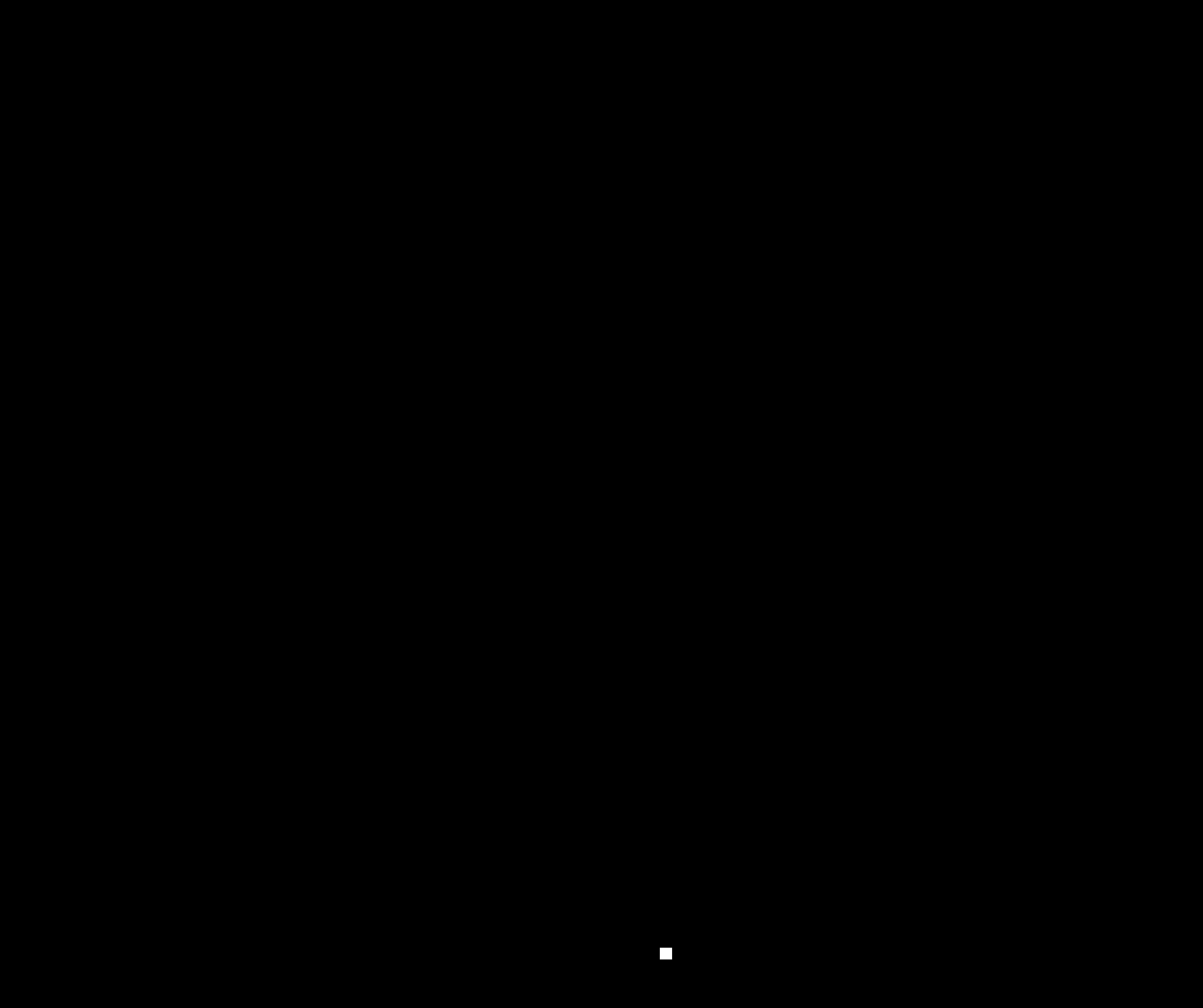

Supplement: Supplemental Information 1 — The supplemental zip file contains 3 folders: data, scripts, and license. The scripts enable denovo analysis of the data contained in the data folder, which was used to generate the figures in the manuscript. The license is GPL version2. [file peerj-06-5727-s001.zip › analysis/data/plant/card_masks/13_mask.png]

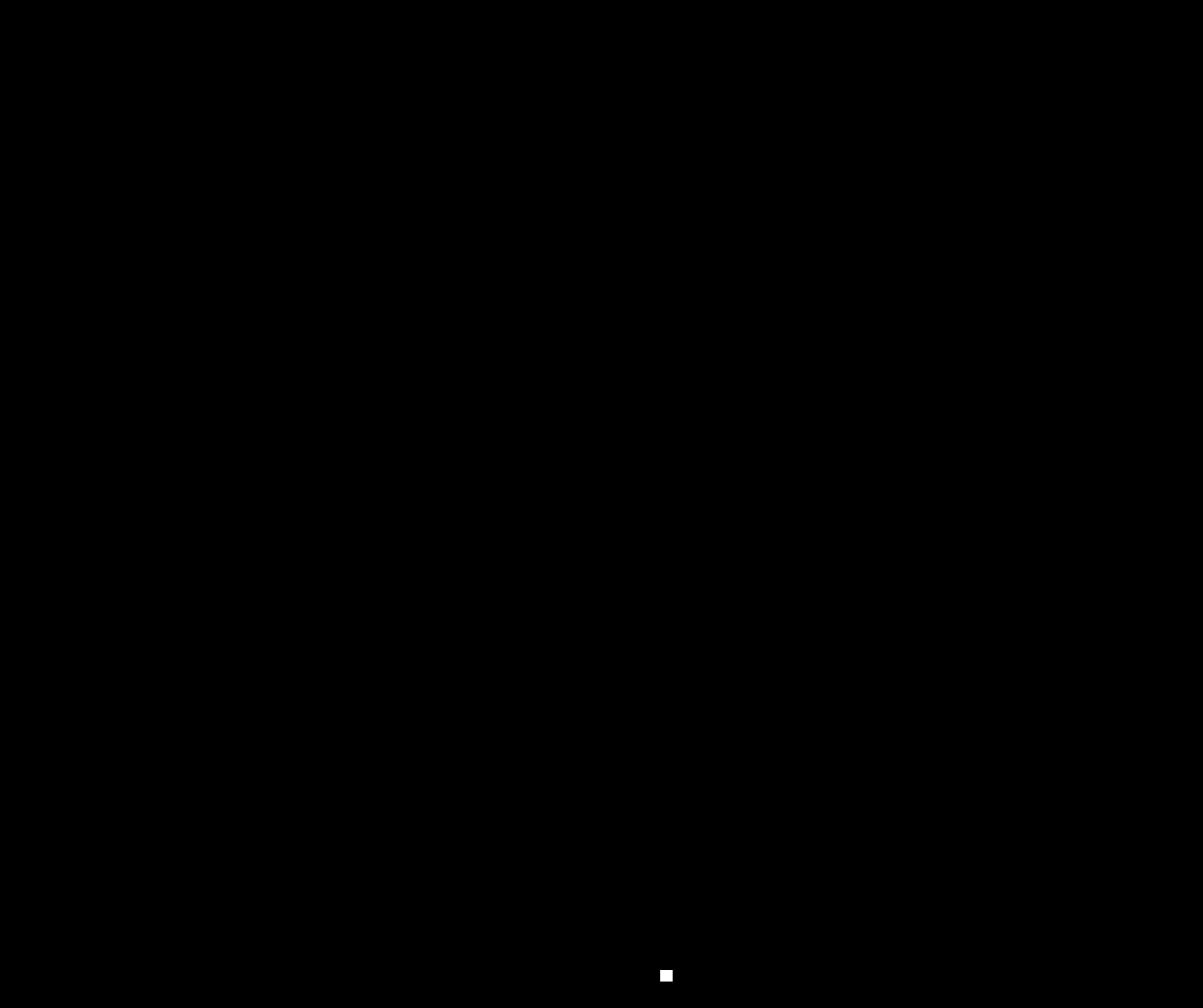

Supplement: Supplemental Information 1 — The supplemental zip file contains 3 folders: data, scripts, and license. The scripts enable denovo analysis of the data contained in the data folder, which was used to generate the figures in the manuscript. The license is GPL version2. [file peerj-06-5727-s001.zip › analysis/data/plant/card_masks/14_mask.png]

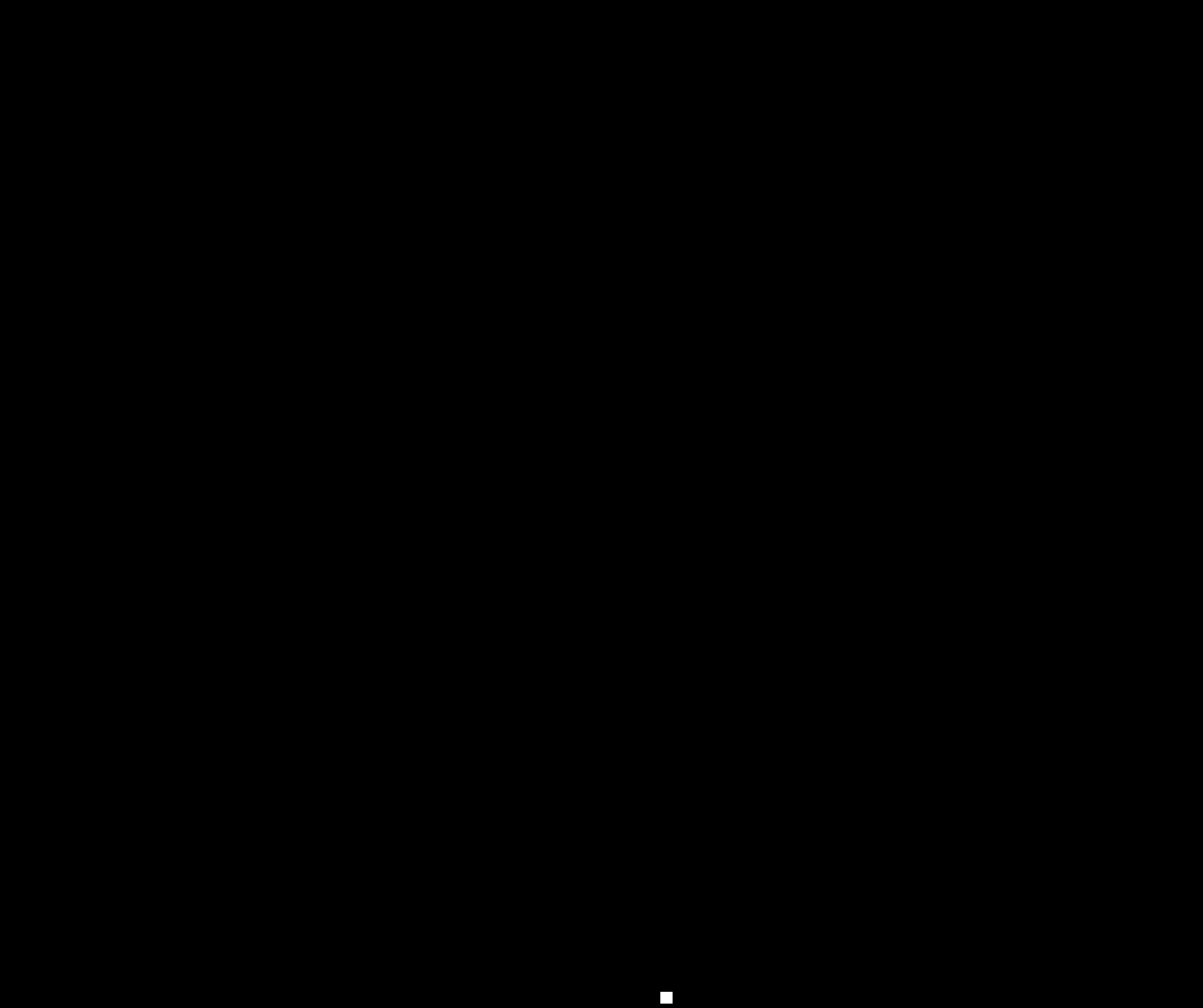

Supplement: Supplemental Information 1 — The supplemental zip file contains 3 folders: data, scripts, and license. The scripts enable denovo analysis of the data contained in the data folder, which was used to generate the figures in the manuscript. The license is GPL version2. [file peerj-06-5727-s001.zip › analysis/data/plant/card_masks/15_mask.png]

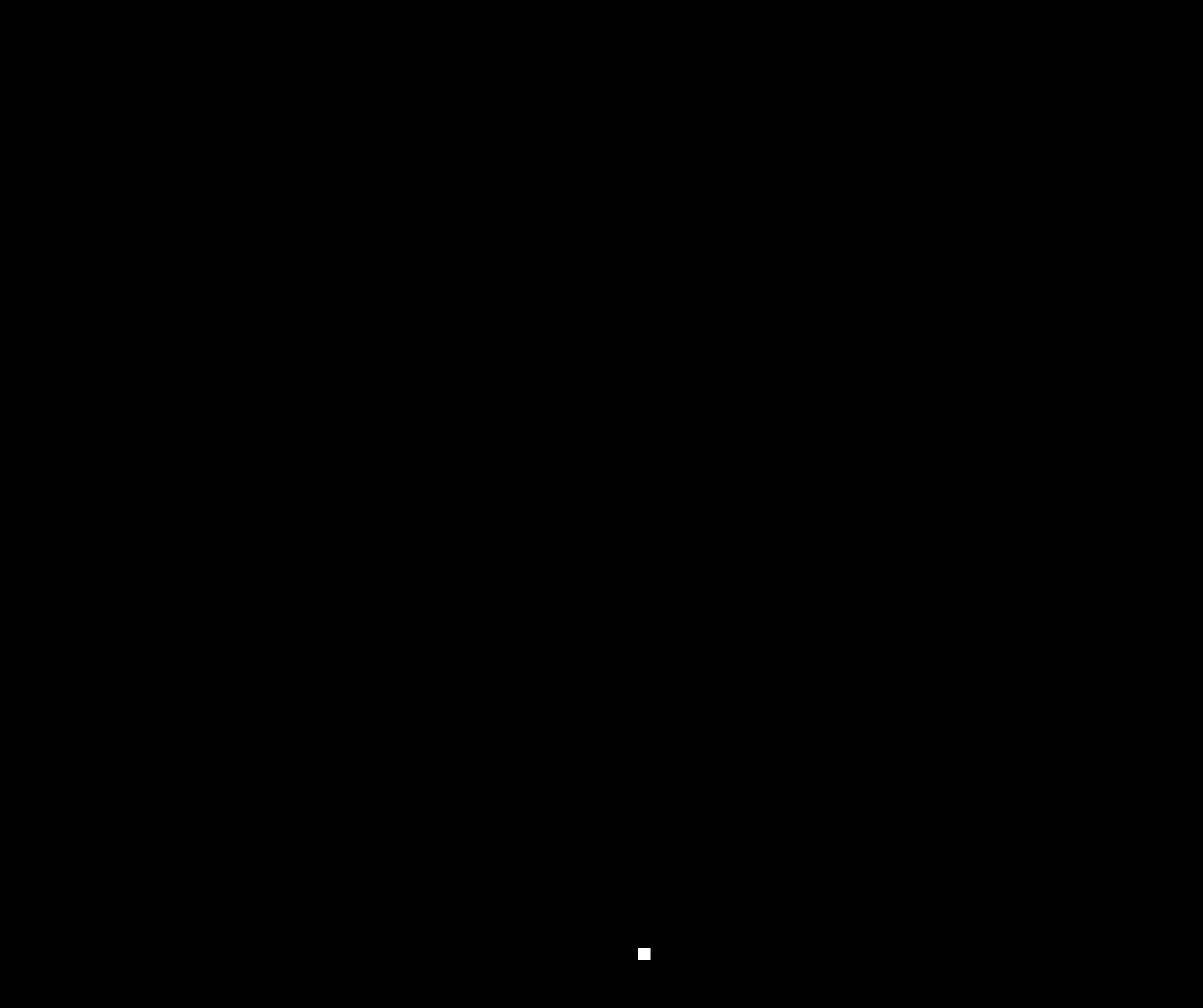

Supplement: Supplemental Information 1 — The supplemental zip file contains 3 folders: data, scripts, and license. The scripts enable denovo analysis of the data contained in the data folder, which was used to generate the figures in the manuscript. The license is GPL version2. [file peerj-06-5727-s001.zip › analysis/data/plant/card_masks/16_mask.png]

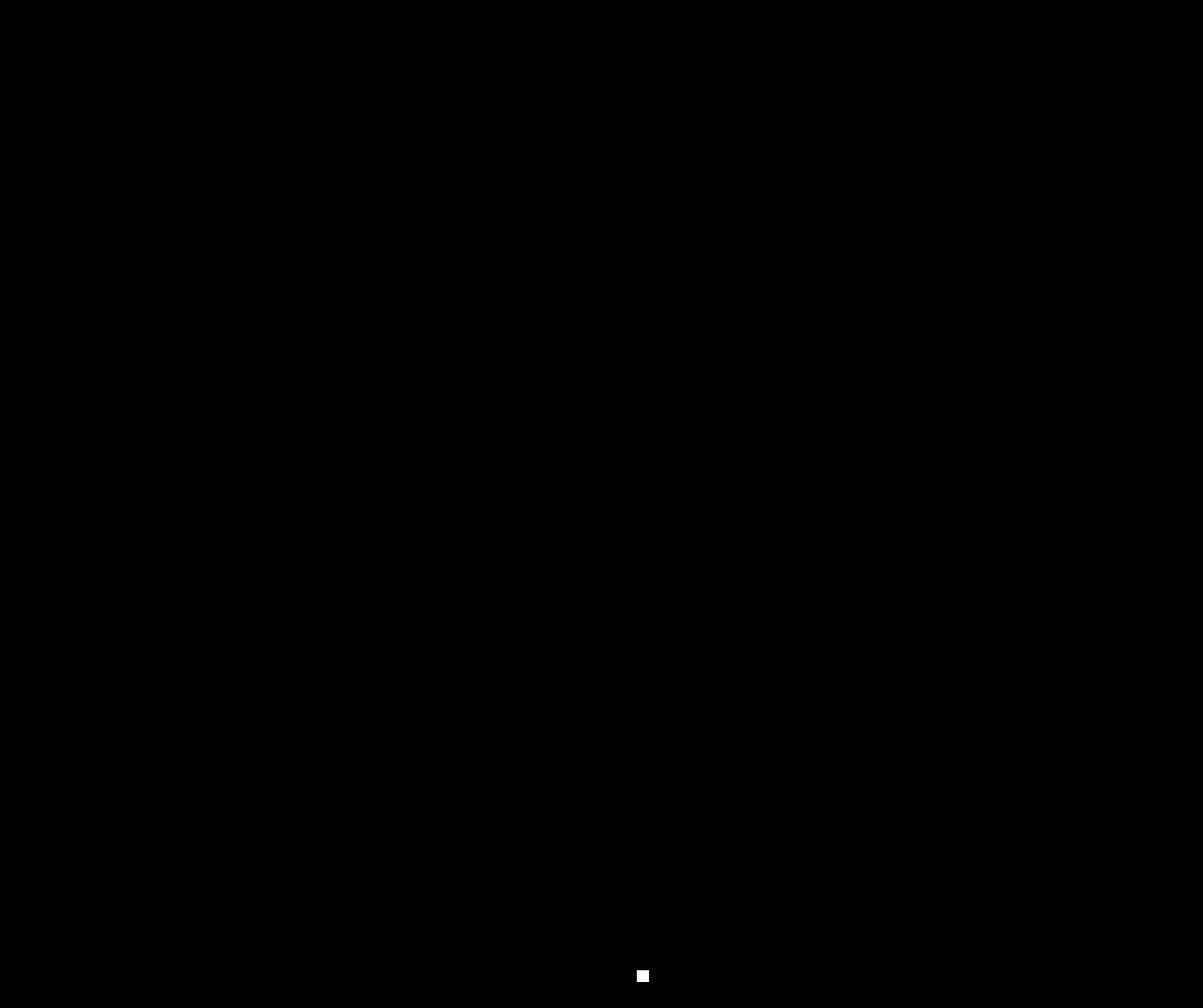

Supplement: Supplemental Information 1 — The supplemental zip file contains 3 folders: data, scripts, and license. The scripts enable denovo analysis of the data contained in the data folder, which was used to generate the figures in the manuscript. The license is GPL version2. [file peerj-06-5727-s001.zip › analysis/data/plant/card_masks/17_mask.png]

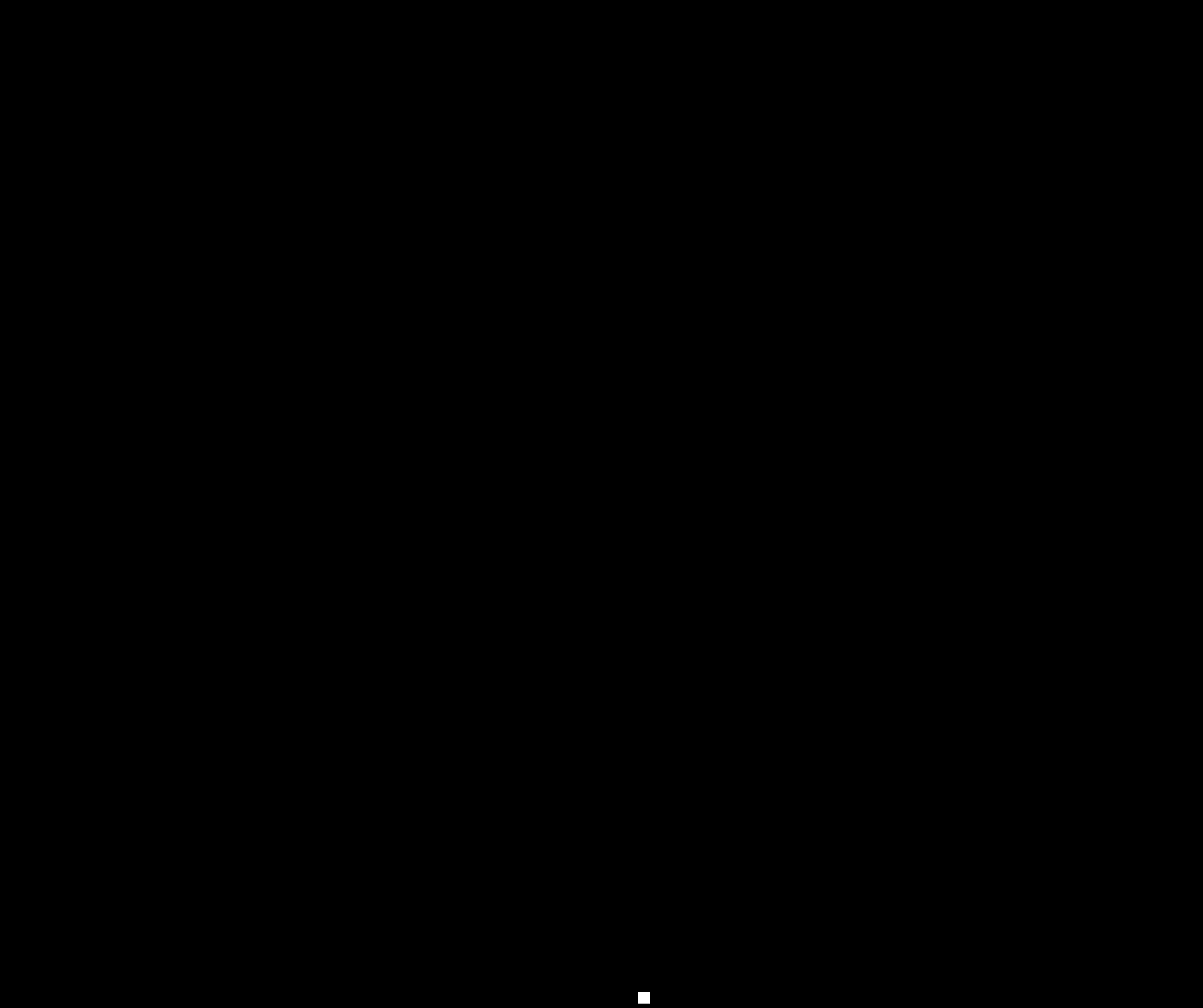

Supplement: Supplemental Information 1 — The supplemental zip file contains 3 folders: data, scripts, and license. The scripts enable denovo analysis of the data contained in the data folder, which was used to generate the figures in the manuscript. The license is GPL version2. [file peerj-06-5727-s001.zip › analysis/data/plant/card_masks/18_mask.png]

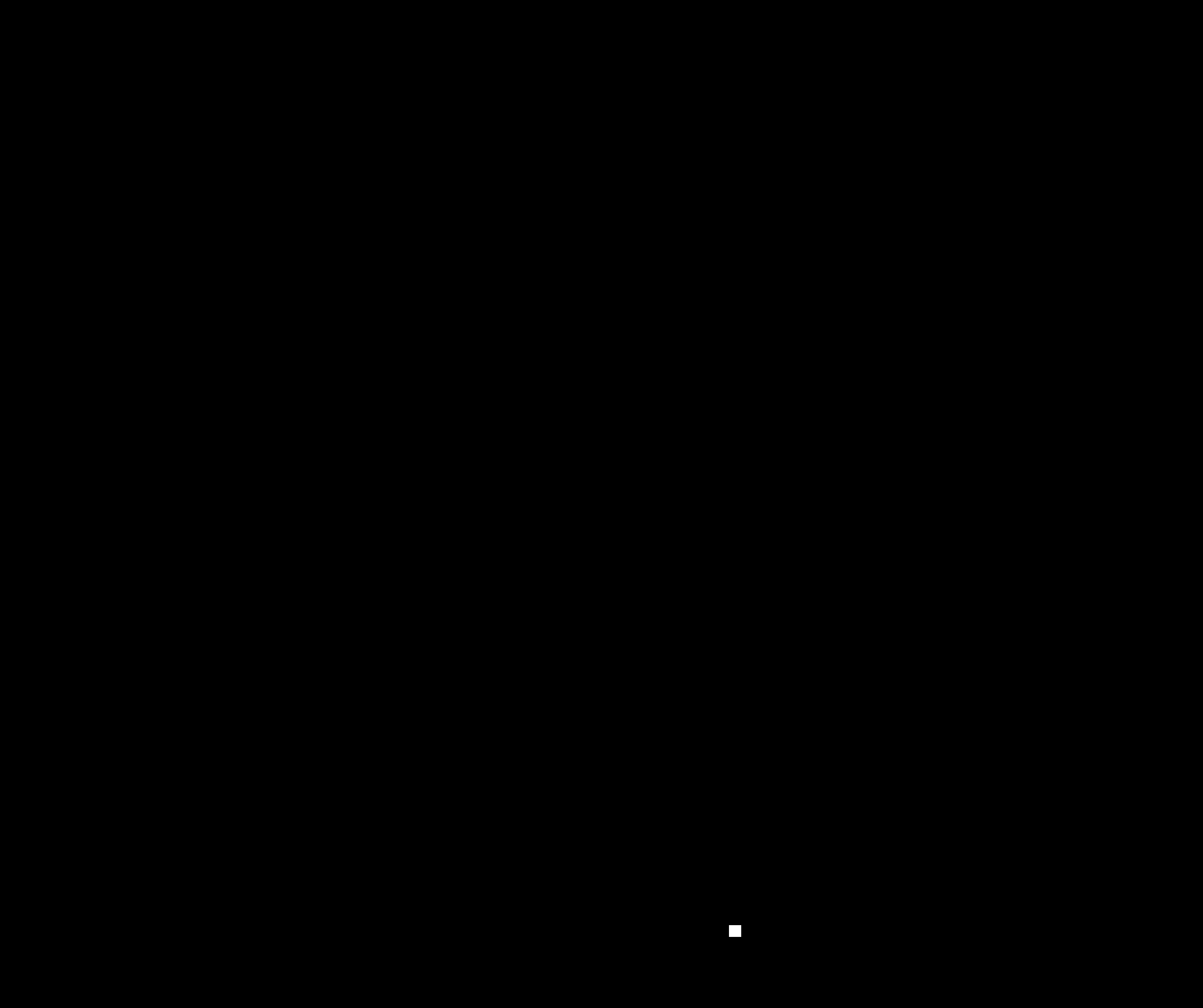

Supplement: Supplemental Information 1 — The supplemental zip file contains 3 folders: data, scripts, and license. The scripts enable denovo analysis of the data contained in the data folder, which was used to generate the figures in the manuscript. The license is GPL version2. [file peerj-06-5727-s001.zip › analysis/data/plant/card_masks/19_mask.png]

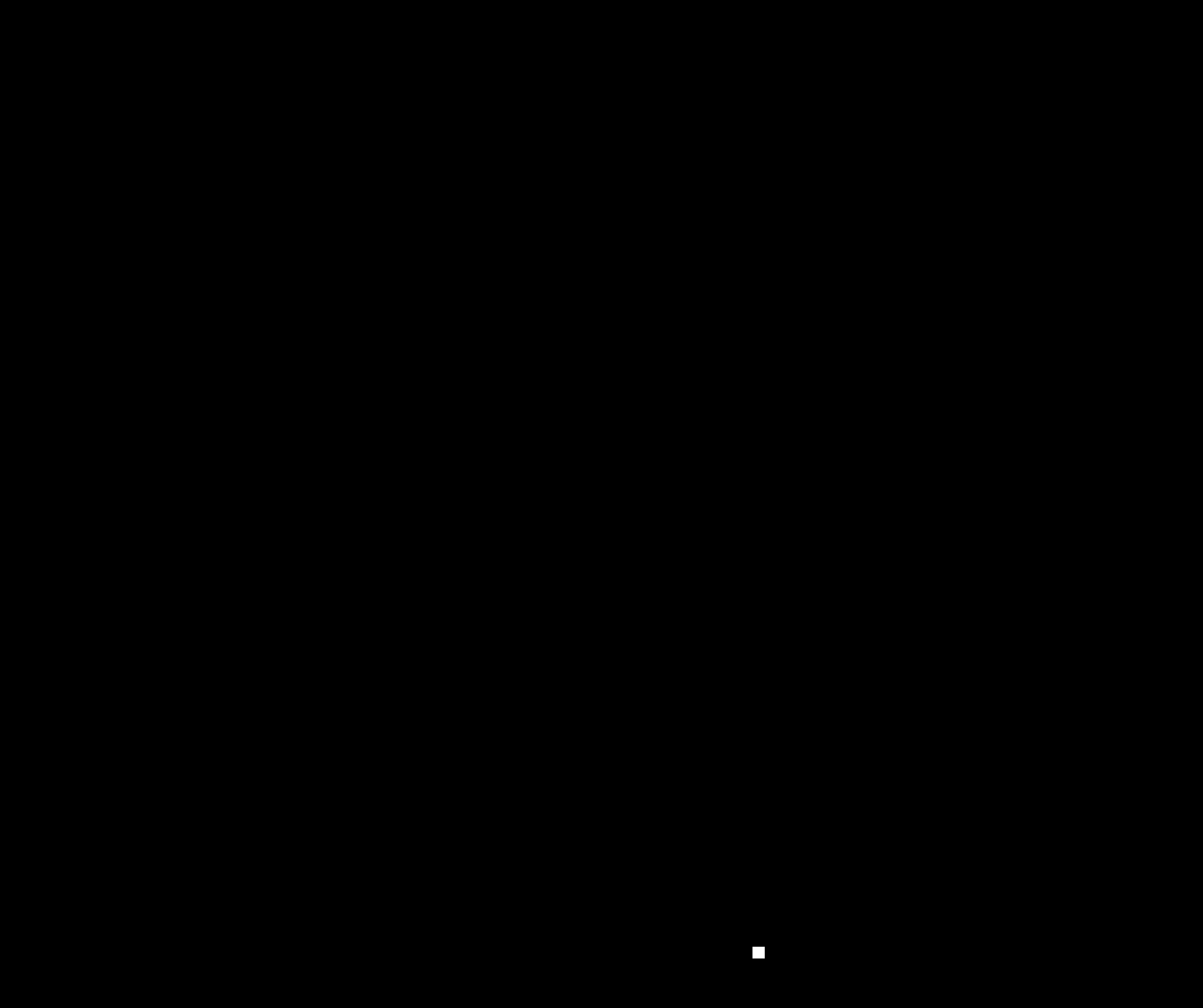

Supplement: Supplemental Information 1 — The supplemental zip file contains 3 folders: data, scripts, and license. The scripts enable denovo analysis of the data contained in the data folder, which was used to generate the figures in the manuscript. The license is GPL version2. [file peerj-06-5727-s001.zip › analysis/data/plant/card_masks/1_mask.png]

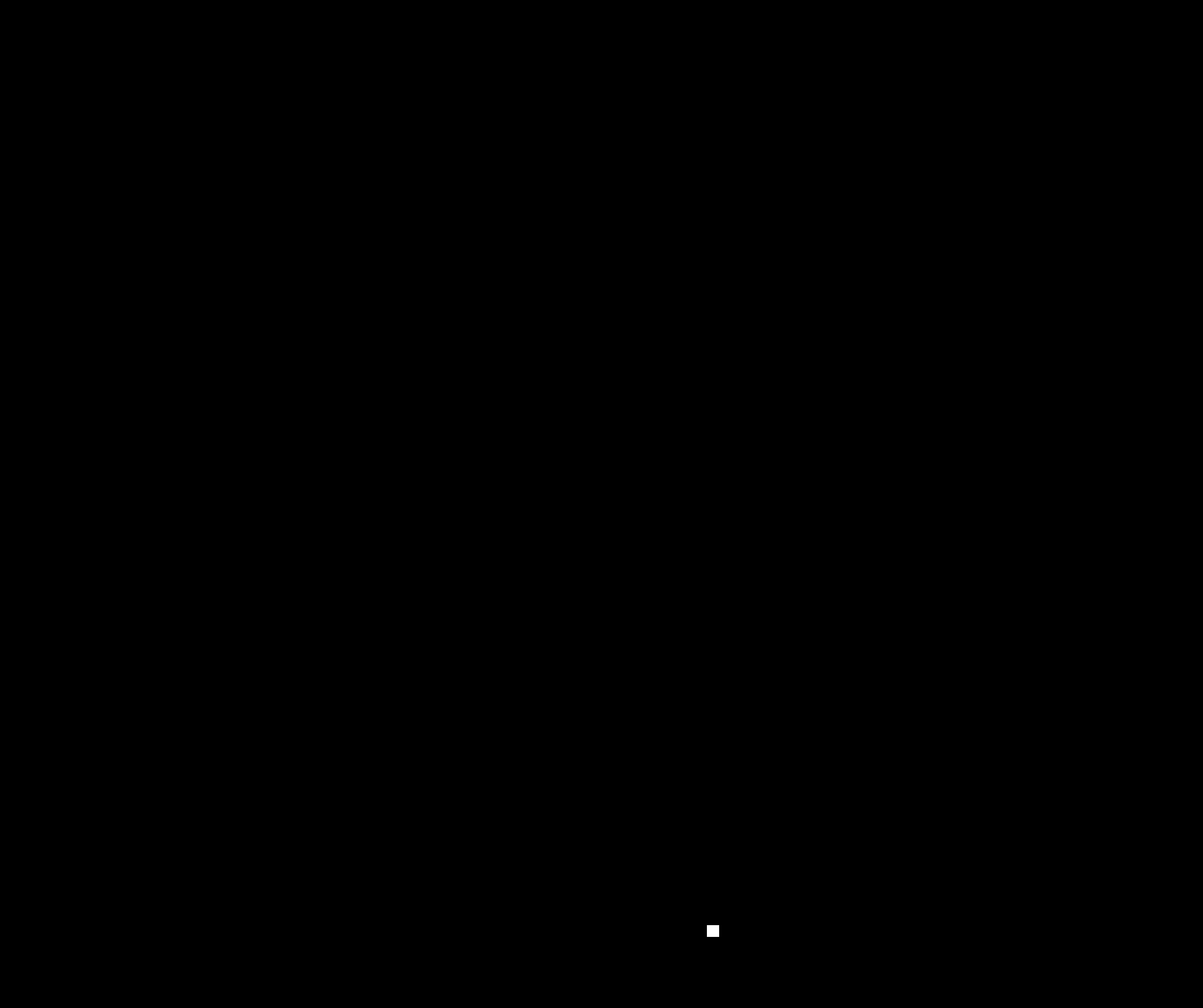

Supplement: Supplemental Information 1 — The supplemental zip file contains 3 folders: data, scripts, and license. The scripts enable denovo analysis of the data contained in the data folder, which was used to generate the figures in the manuscript. The license is GPL version2. [file peerj-06-5727-s001.zip › analysis/data/plant/card_masks/20_mask.png]

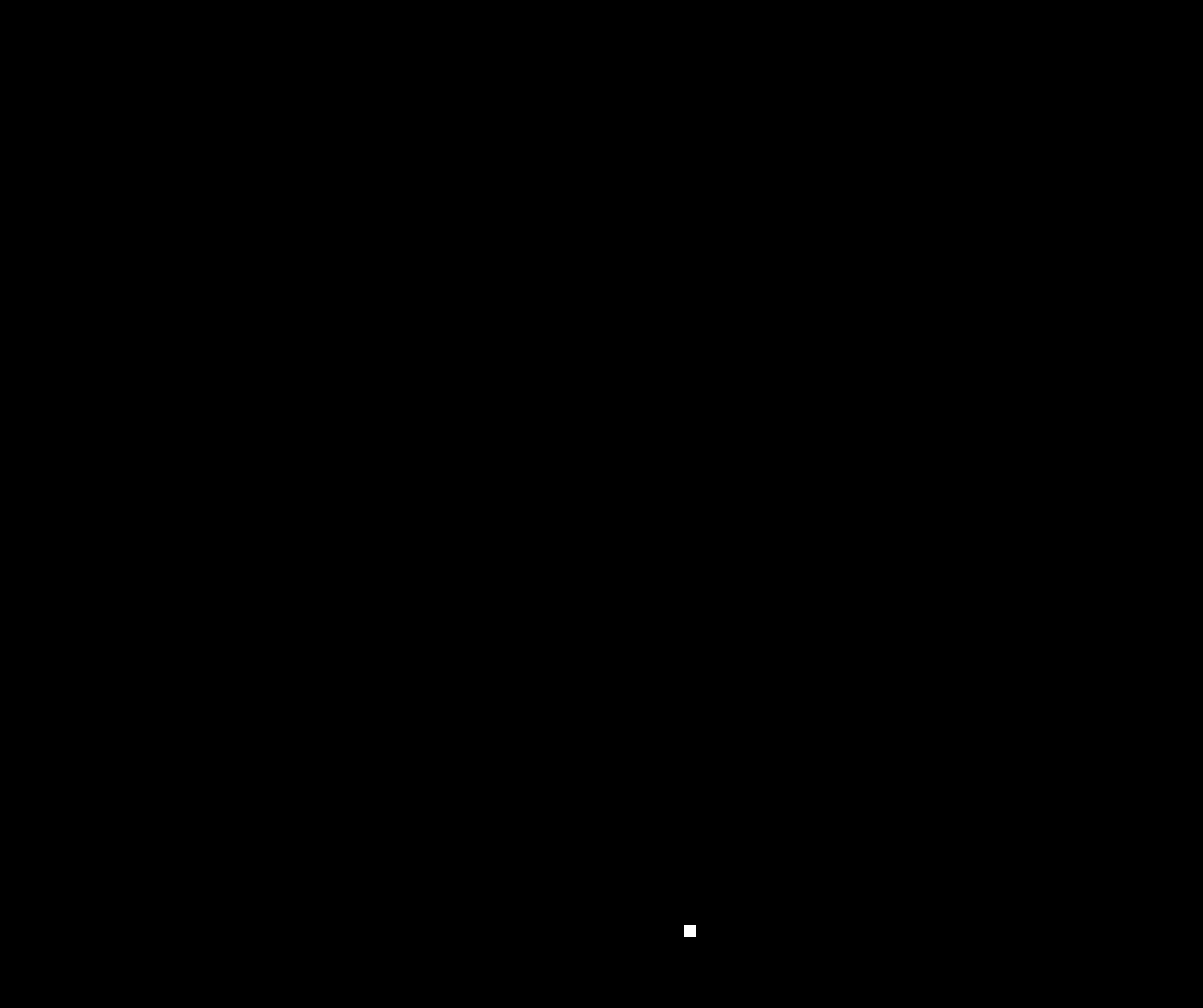

Supplement: Supplemental Information 1 — The supplemental zip file contains 3 folders: data, scripts, and license. The scripts enable denovo analysis of the data contained in the data folder, which was used to generate the figures in the manuscript. The license is GPL version2. [file peerj-06-5727-s001.zip › analysis/data/plant/card_masks/21_mask.png]

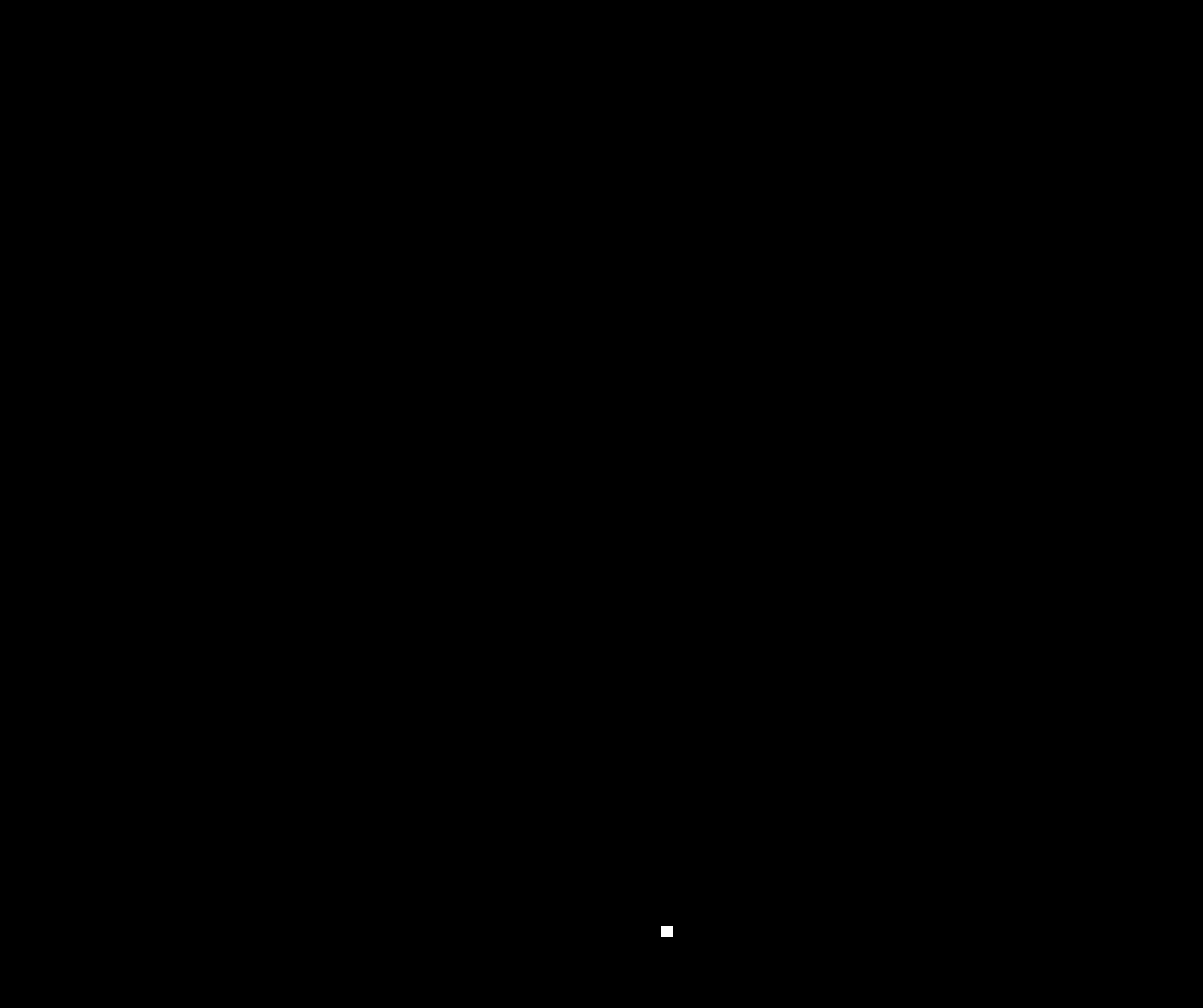

Supplement: Supplemental Information 1 — The supplemental zip file contains 3 folders: data, scripts, and license. The scripts enable denovo analysis of the data contained in the data folder, which was used to generate the figures in the manuscript. The license is GPL version2. [file peerj-06-5727-s001.zip › analysis/data/plant/card_masks/22_mask.png]

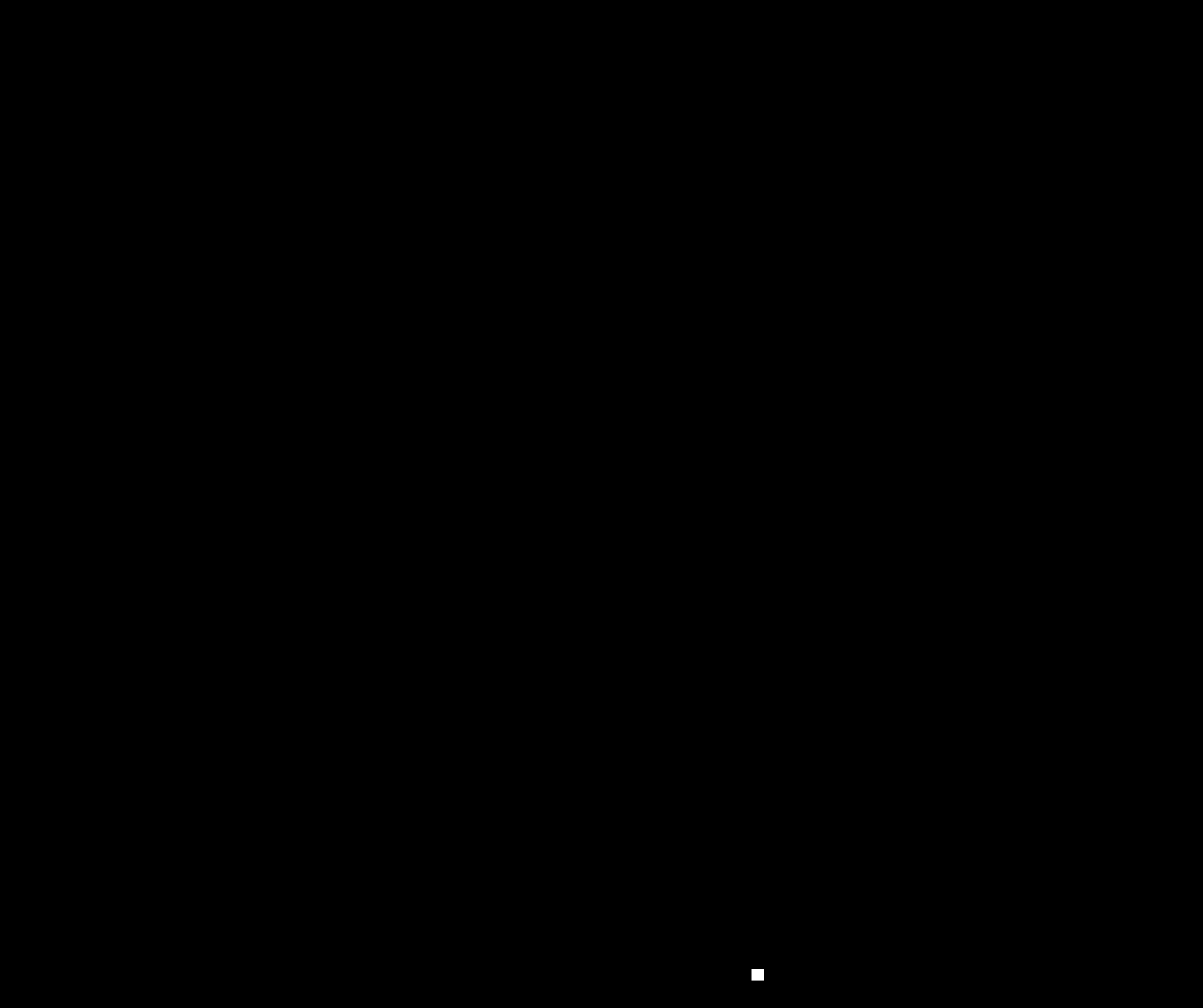

Supplement: Supplemental Information 1 — The supplemental zip file contains 3 folders: data, scripts, and license. The scripts enable denovo analysis of the data contained in the data folder, which was used to generate the figures in the manuscript. The license is GPL version2. [file peerj-06-5727-s001.zip › analysis/data/plant/card_masks/2_mask.png]

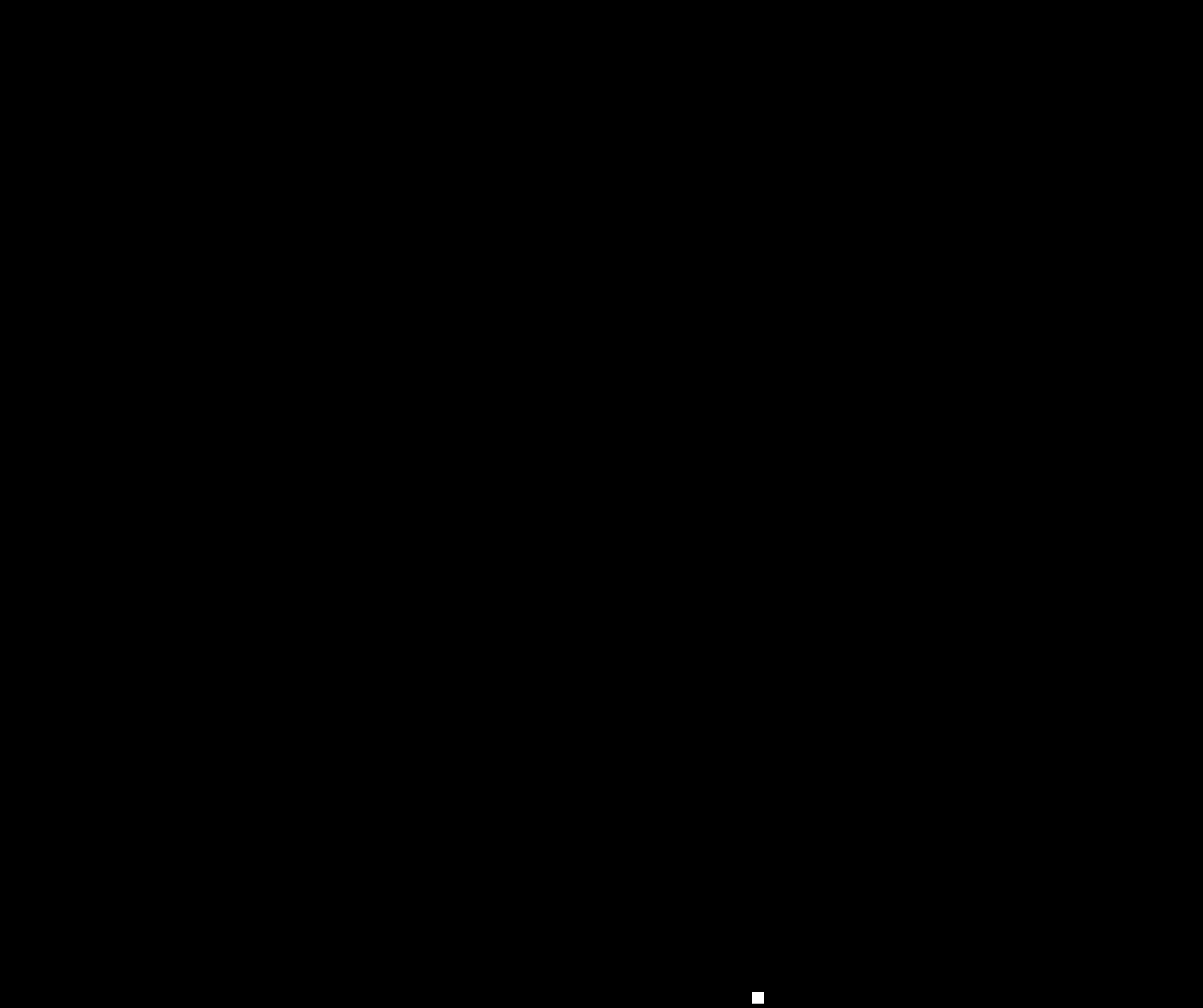

Supplement: Supplemental Information 1 — The supplemental zip file contains 3 folders: data, scripts, and license. The scripts enable denovo analysis of the data contained in the data folder, which was used to generate the figures in the manuscript. The license is GPL version2. [file peerj-06-5727-s001.zip › analysis/data/plant/card_masks/3_mask.png]

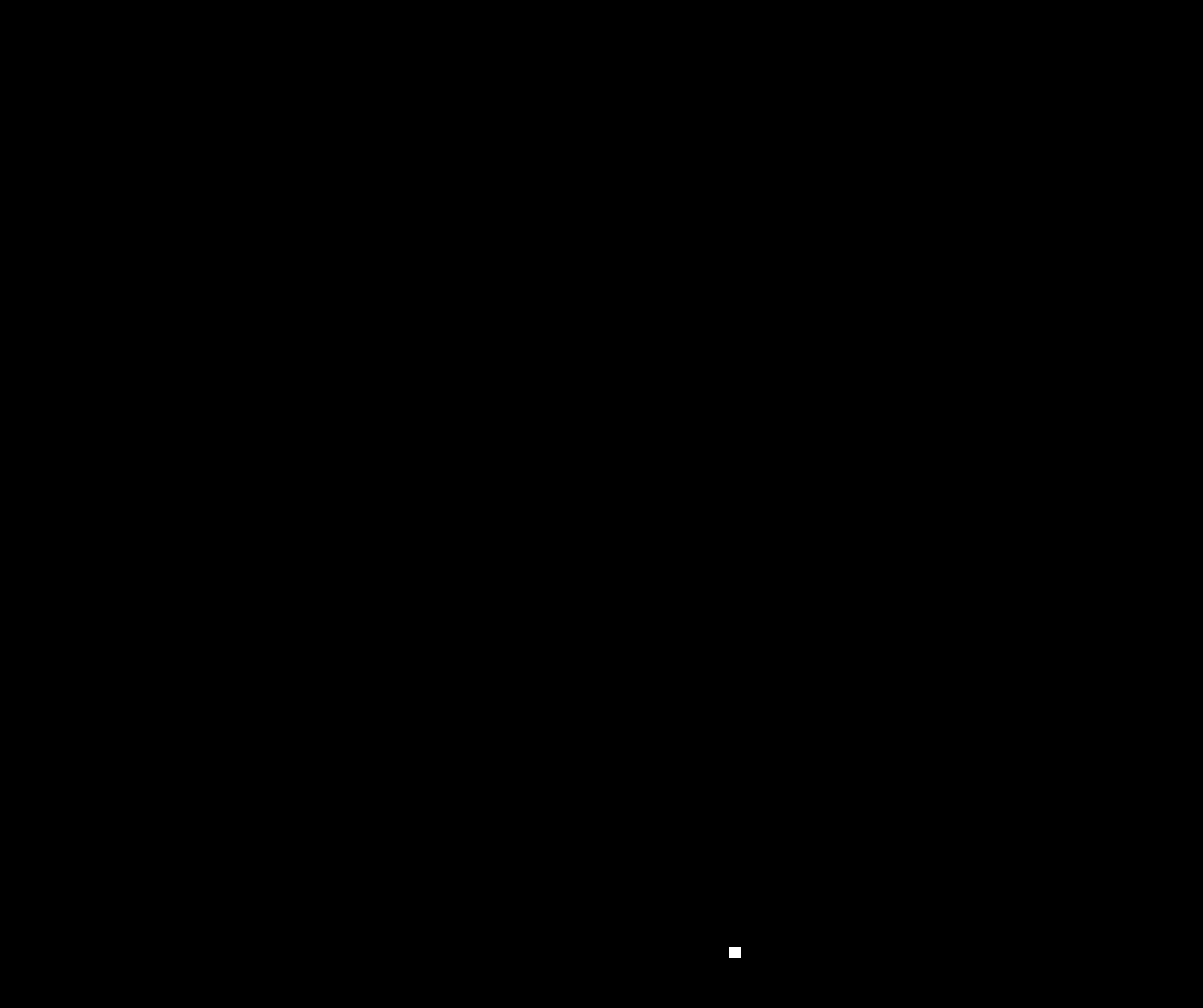

Supplement: Supplemental Information 1 — The supplemental zip file contains 3 folders: data, scripts, and license. The scripts enable denovo analysis of the data contained in the data folder, which was used to generate the figures in the manuscript. The license is GPL version2. [file peerj-06-5727-s001.zip › analysis/data/plant/card_masks/4_mask.png]

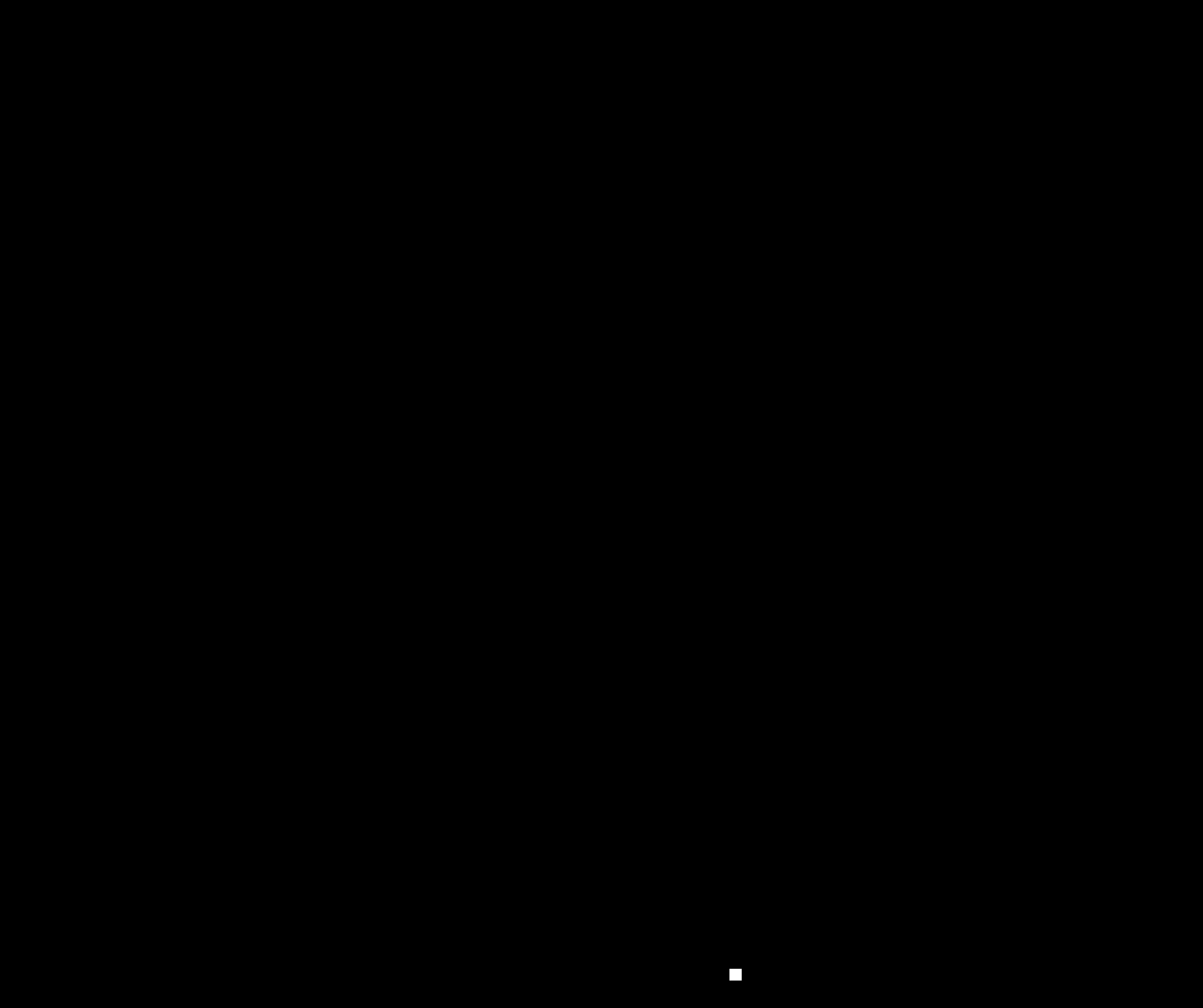

Supplement: Supplemental Information 1 — The supplemental zip file contains 3 folders: data, scripts, and license. The scripts enable denovo analysis of the data contained in the data folder, which was used to generate the figures in the manuscript. The license is GPL version2. [file peerj-06-5727-s001.zip › analysis/data/plant/card_masks/5_mask.png]

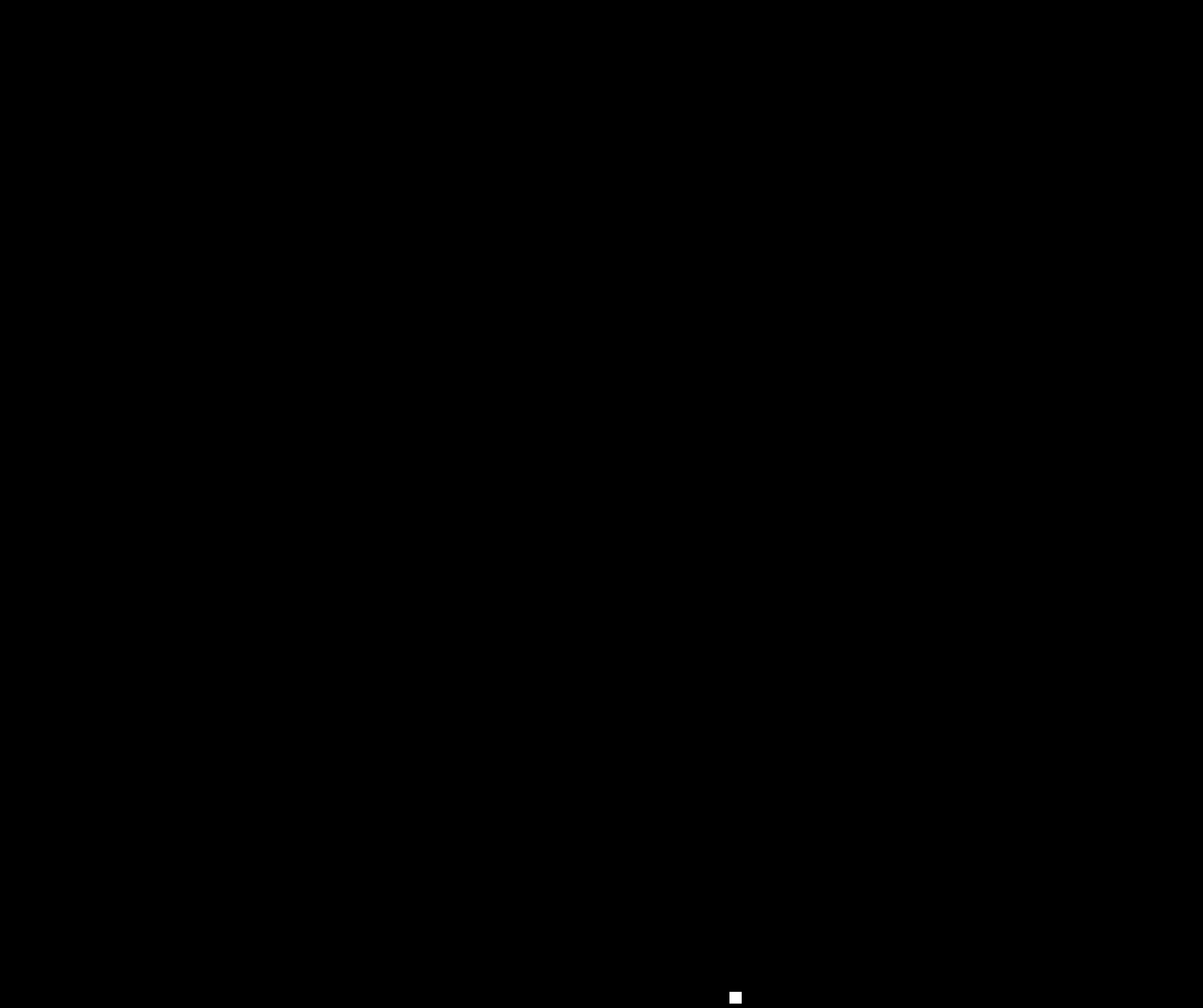

Supplement: Supplemental Information 1 — The supplemental zip file contains 3 folders: data, scripts, and license. The scripts enable denovo analysis of the data contained in the data folder, which was used to generate the figures in the manuscript. The license is GPL version2. [file peerj-06-5727-s001.zip › analysis/data/plant/card_masks/6_mask.png]

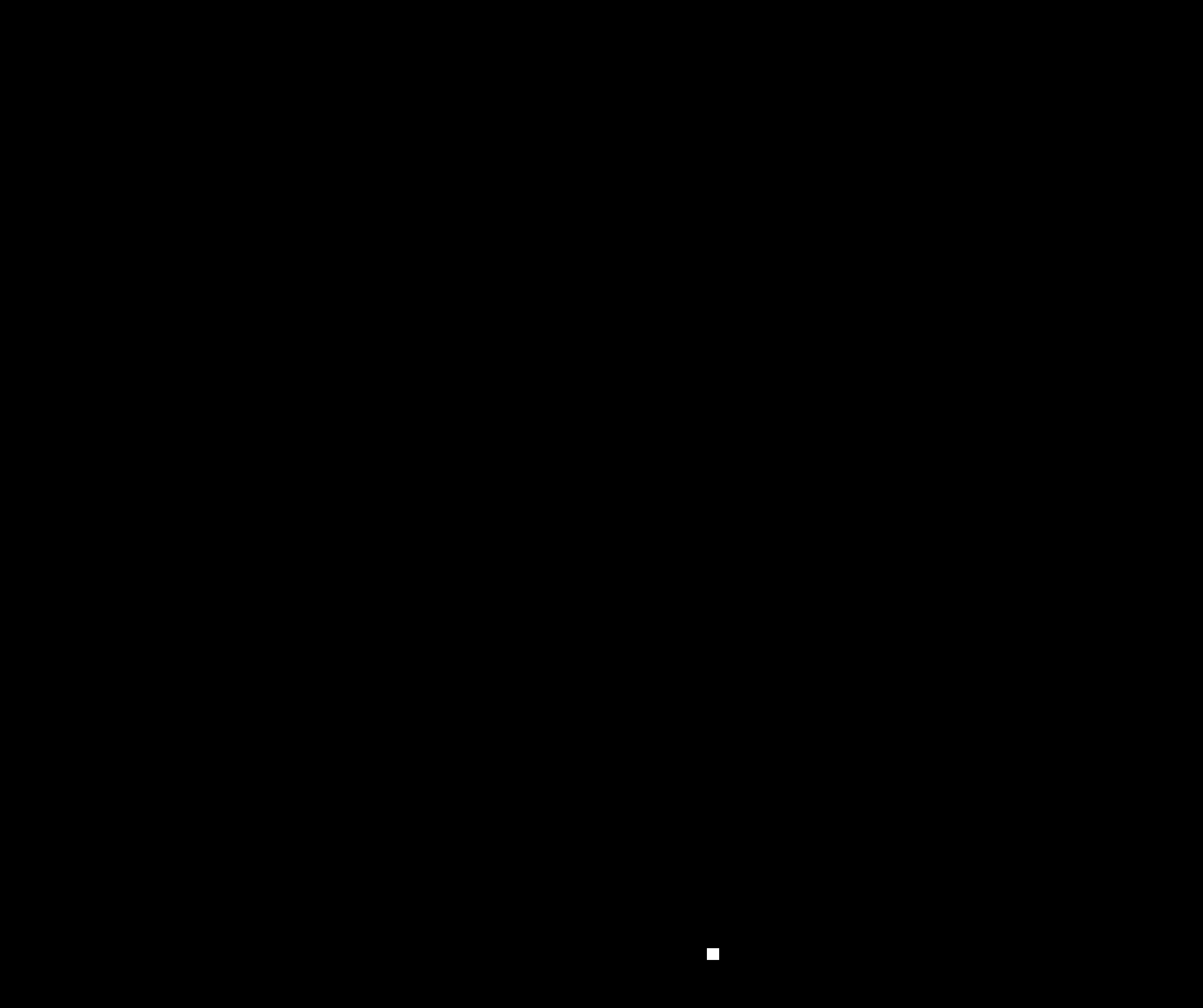

Supplement: Supplemental Information 1 — The supplemental zip file contains 3 folders: data, scripts, and license. The scripts enable denovo analysis of the data contained in the data folder, which was used to generate the figures in the manuscript. The license is GPL version2. [file peerj-06-5727-s001.zip › analysis/data/plant/card_masks/7_mask.png]

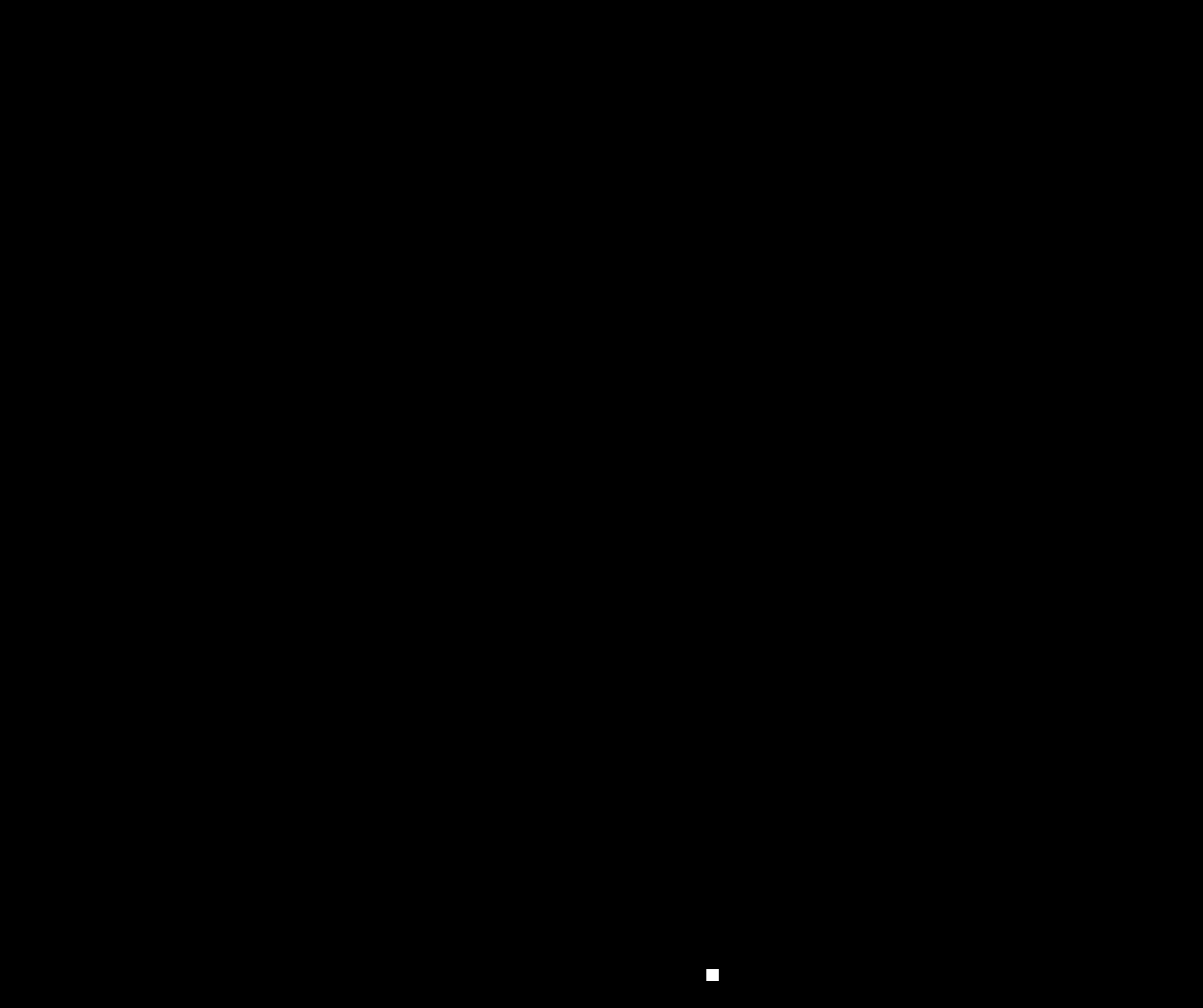

Supplement: Supplemental Information 1 — The supplemental zip file contains 3 folders: data, scripts, and license. The scripts enable denovo analysis of the data contained in the data folder, which was used to generate the figures in the manuscript. The license is GPL version2. [file peerj-06-5727-s001.zip › analysis/data/plant/card_masks/8_mask.png]

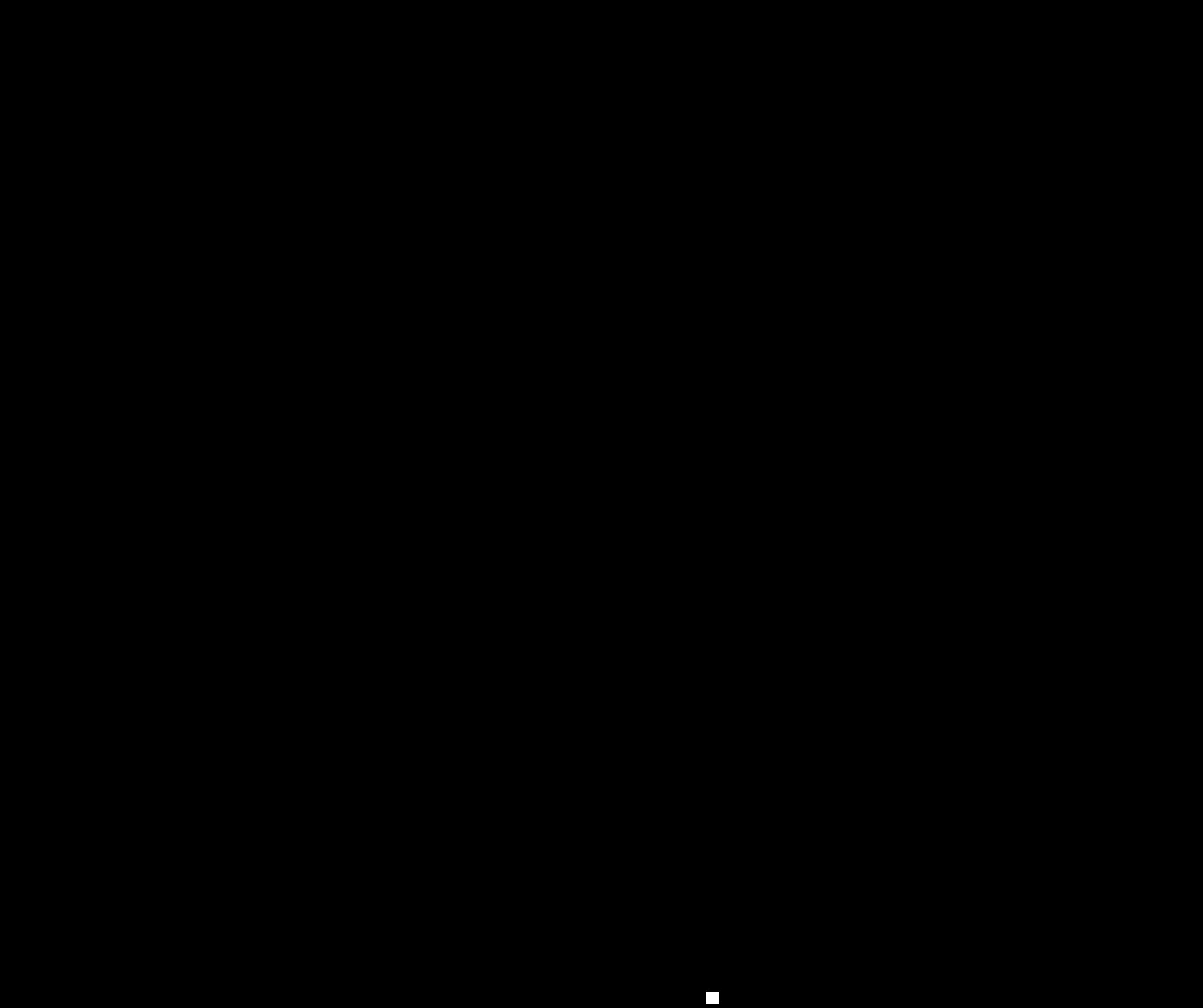

Supplement: Supplemental Information 1 — The supplemental zip file contains 3 folders: data, scripts, and license. The scripts enable denovo analysis of the data contained in the data folder, which was used to generate the figures in the manuscript. The license is GPL version2. [file peerj-06-5727-s001.zip › analysis/data/plant/card_masks/9_mask.png]

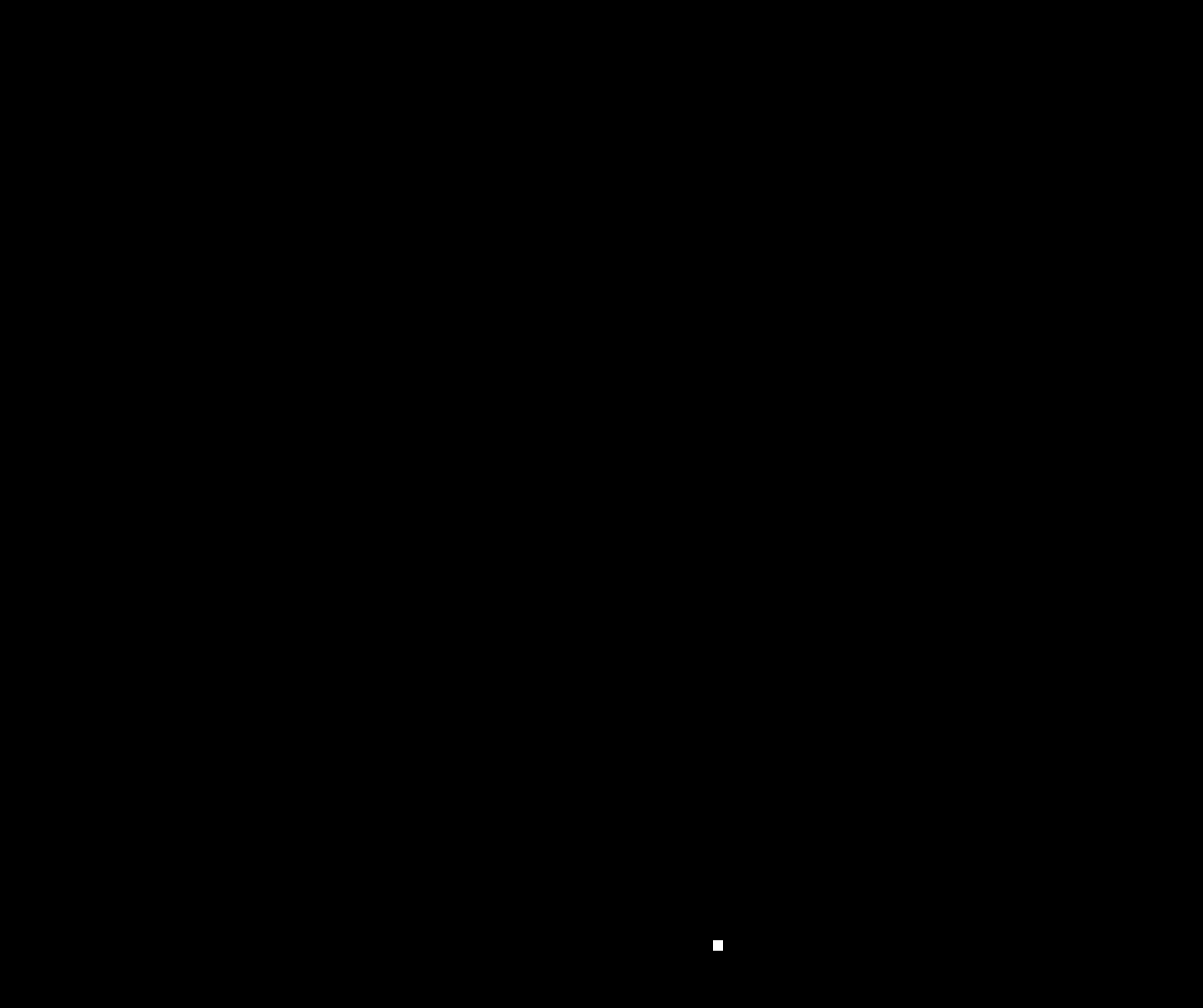

Supplement: Supplemental Information 1 — The supplemental zip file contains 3 folders: data, scripts, and license. The scripts enable denovo analysis of the data contained in the data folder, which was used to generate the figures in the manuscript. The license is GPL version2. [file peerj-06-5727-s001.zip › analysis/data/temperature/card_masks/10_mask.png]

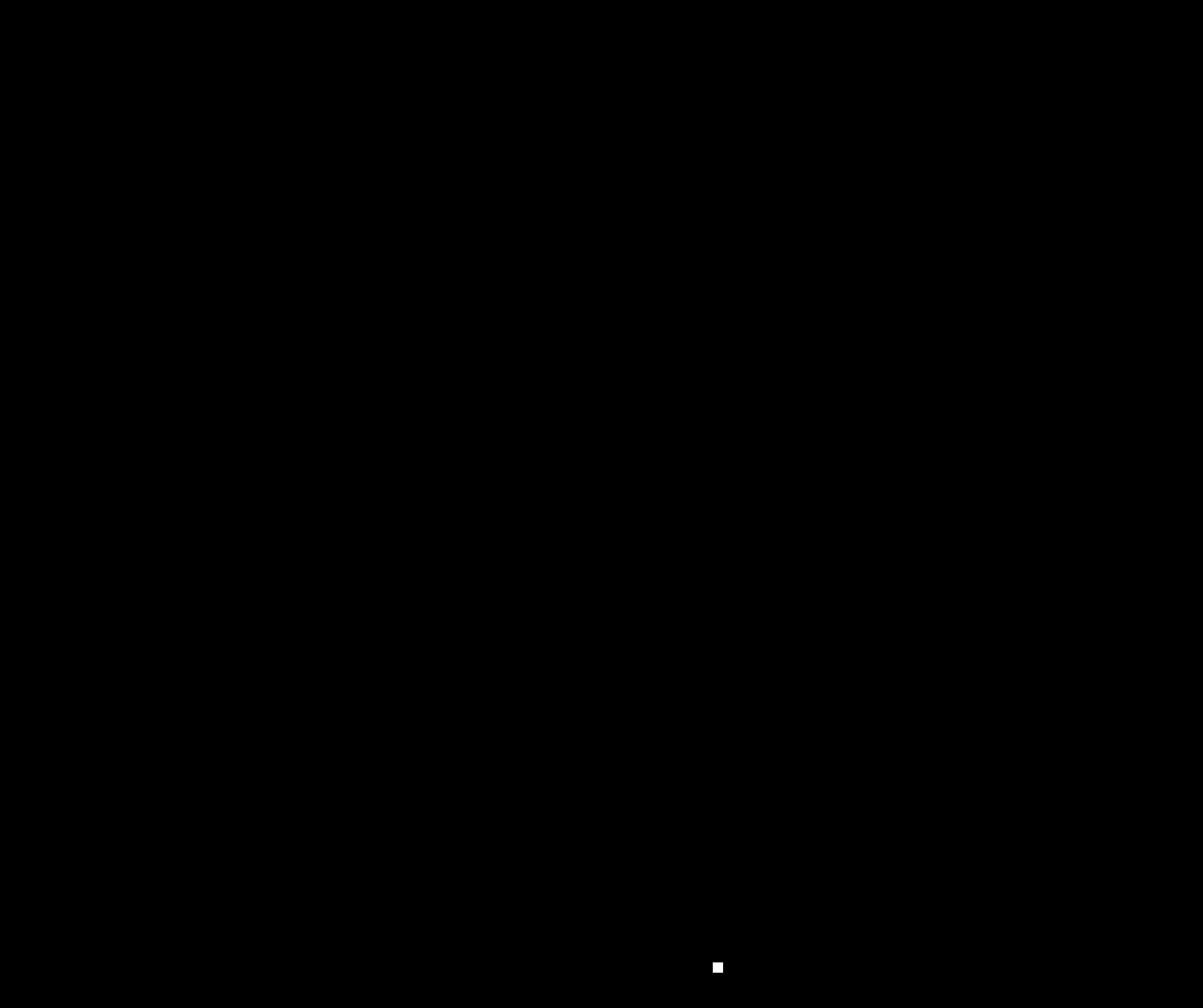

Supplement: Supplemental Information 1 — The supplemental zip file contains 3 folders: data, scripts, and license. The scripts enable denovo analysis of the data contained in the data folder, which was used to generate the figures in the manuscript. The license is GPL version2. [file peerj-06-5727-s001.zip › analysis/data/temperature/card_masks/11_mask.png]

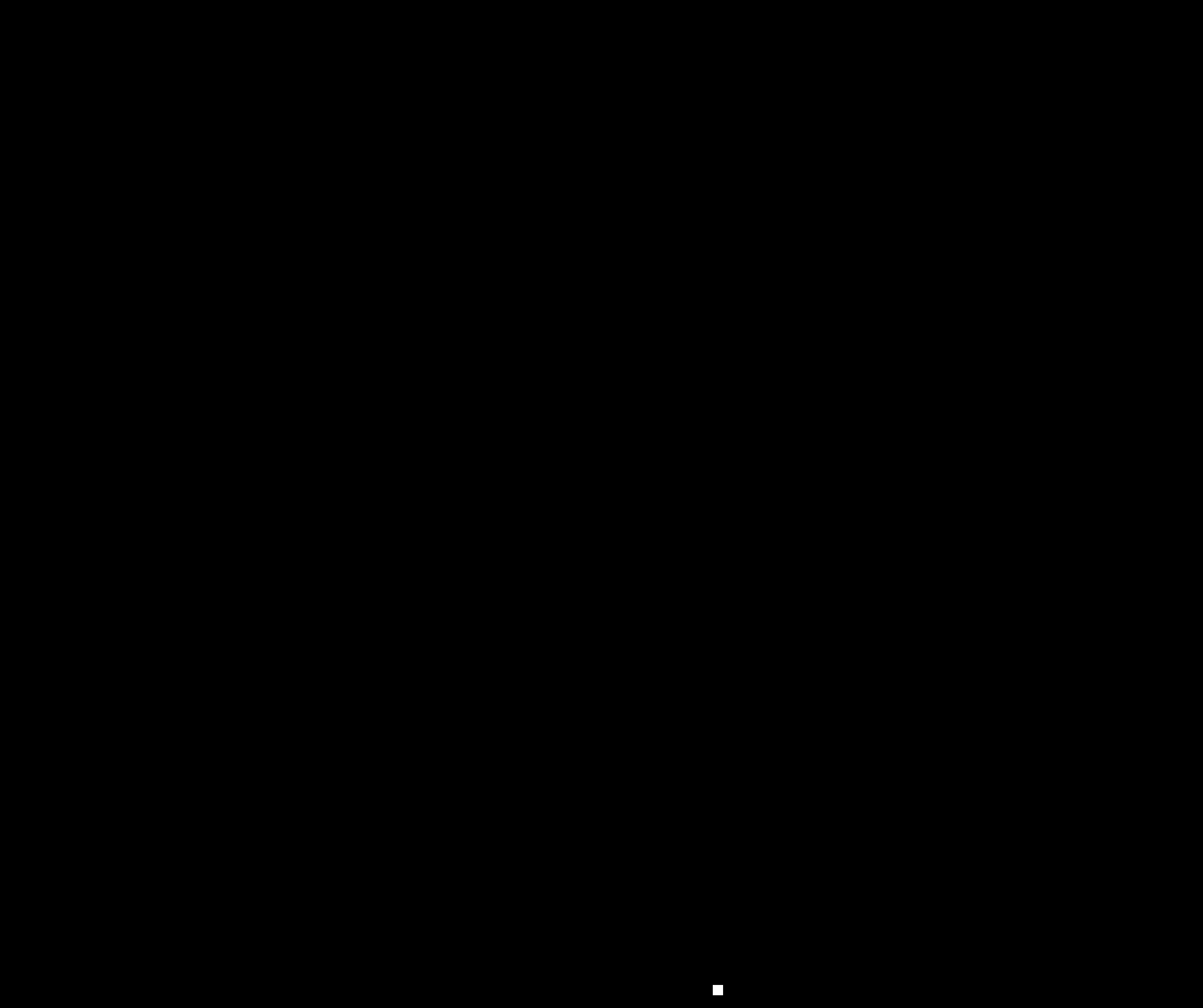

Supplement: Supplemental Information 1 — The supplemental zip file contains 3 folders: data, scripts, and license. The scripts enable denovo analysis of the data contained in the data folder, which was used to generate the figures in the manuscript. The license is GPL version2. [file peerj-06-5727-s001.zip › analysis/data/temperature/card_masks/12_mask.png]

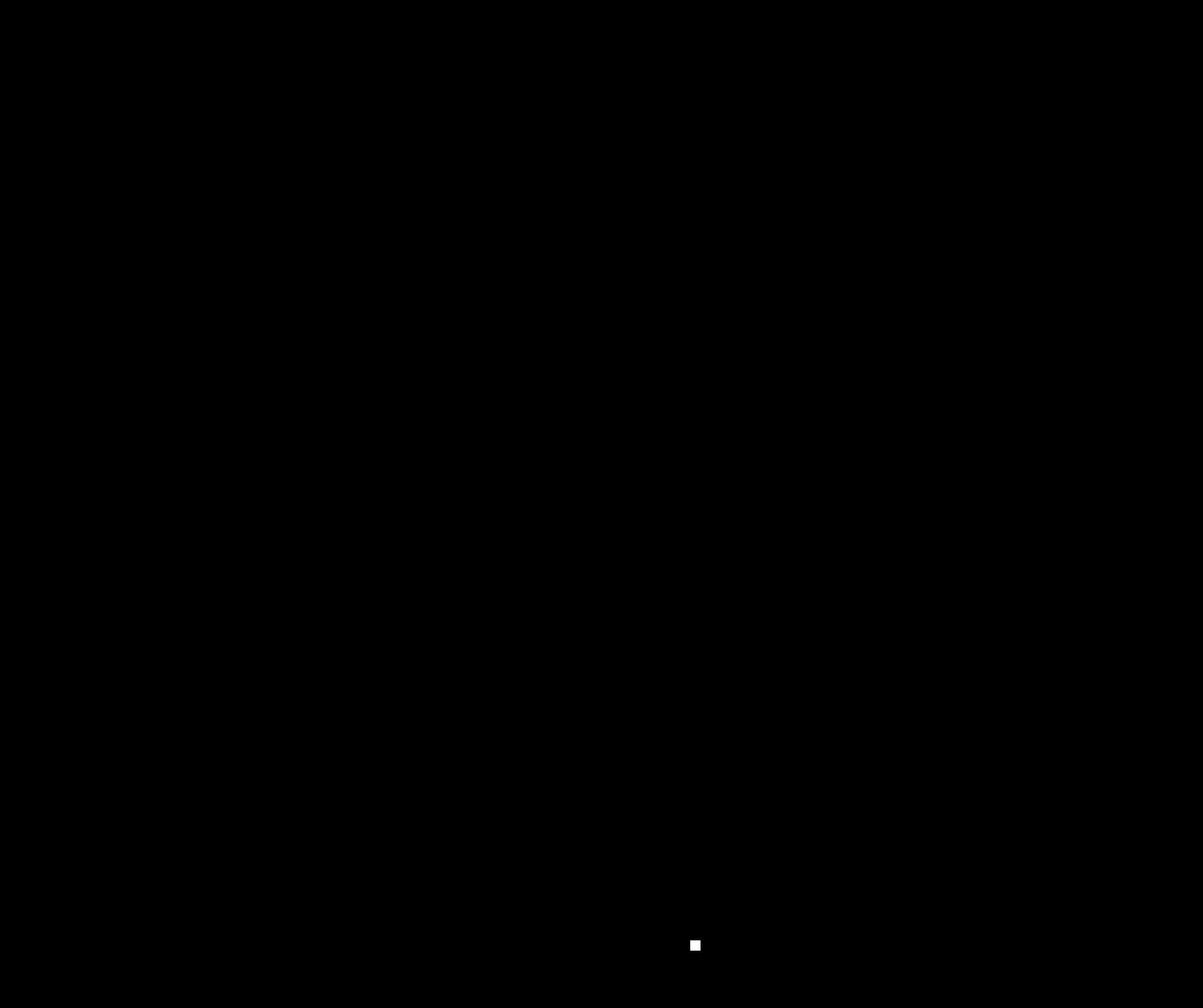

Supplement: Supplemental Information 1 — The supplemental zip file contains 3 folders: data, scripts, and license. The scripts enable denovo analysis of the data contained in the data folder, which was used to generate the figures in the manuscript. The license is GPL version2. [file peerj-06-5727-s001.zip › analysis/data/temperature/card_masks/13_mask.png]

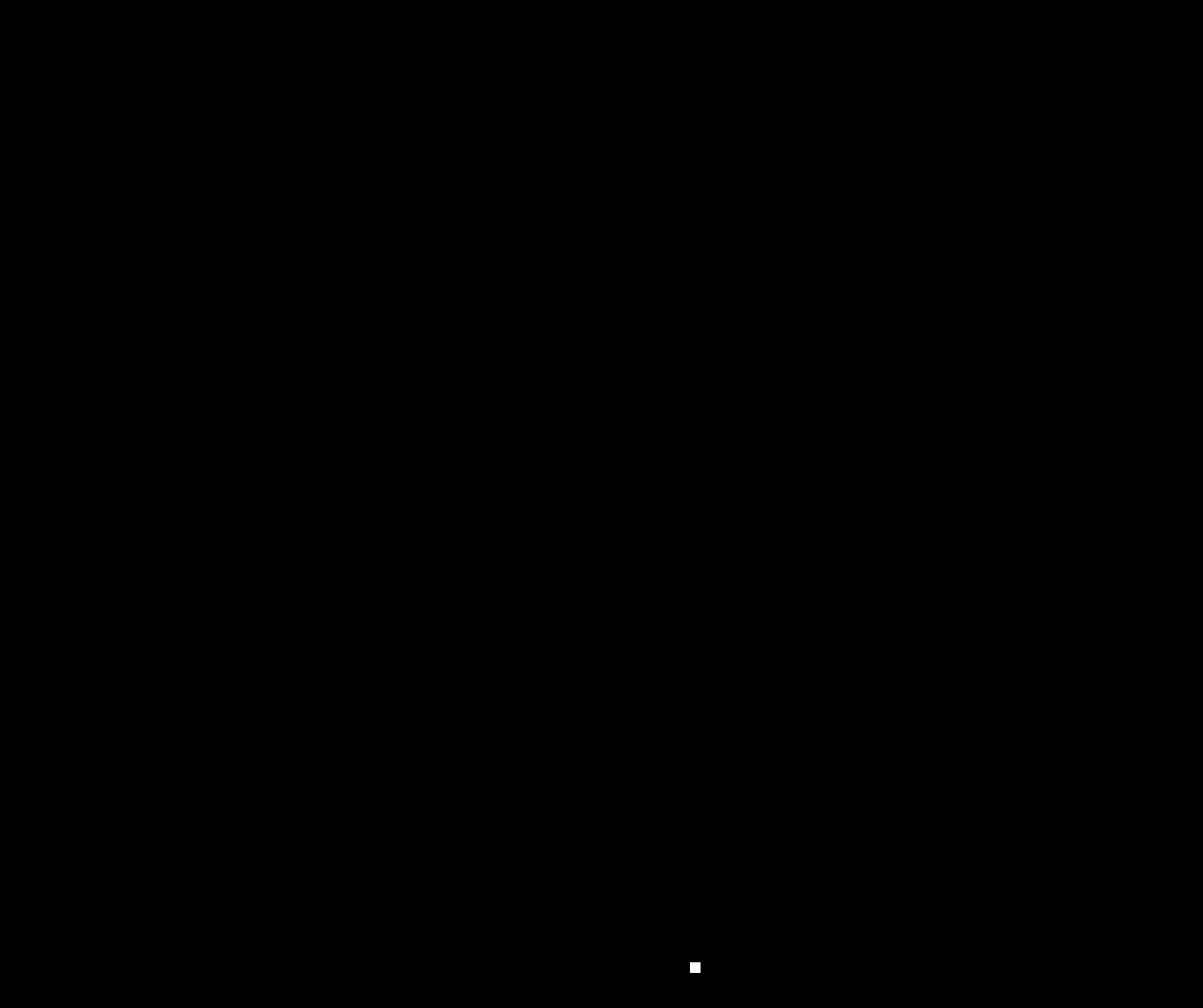

Supplement: Supplemental Information 1 — The supplemental zip file contains 3 folders: data, scripts, and license. The scripts enable denovo analysis of the data contained in the data folder, which was used to generate the figures in the manuscript. The license is GPL version2. [file peerj-06-5727-s001.zip › analysis/data/temperature/card_masks/14_mask.png]

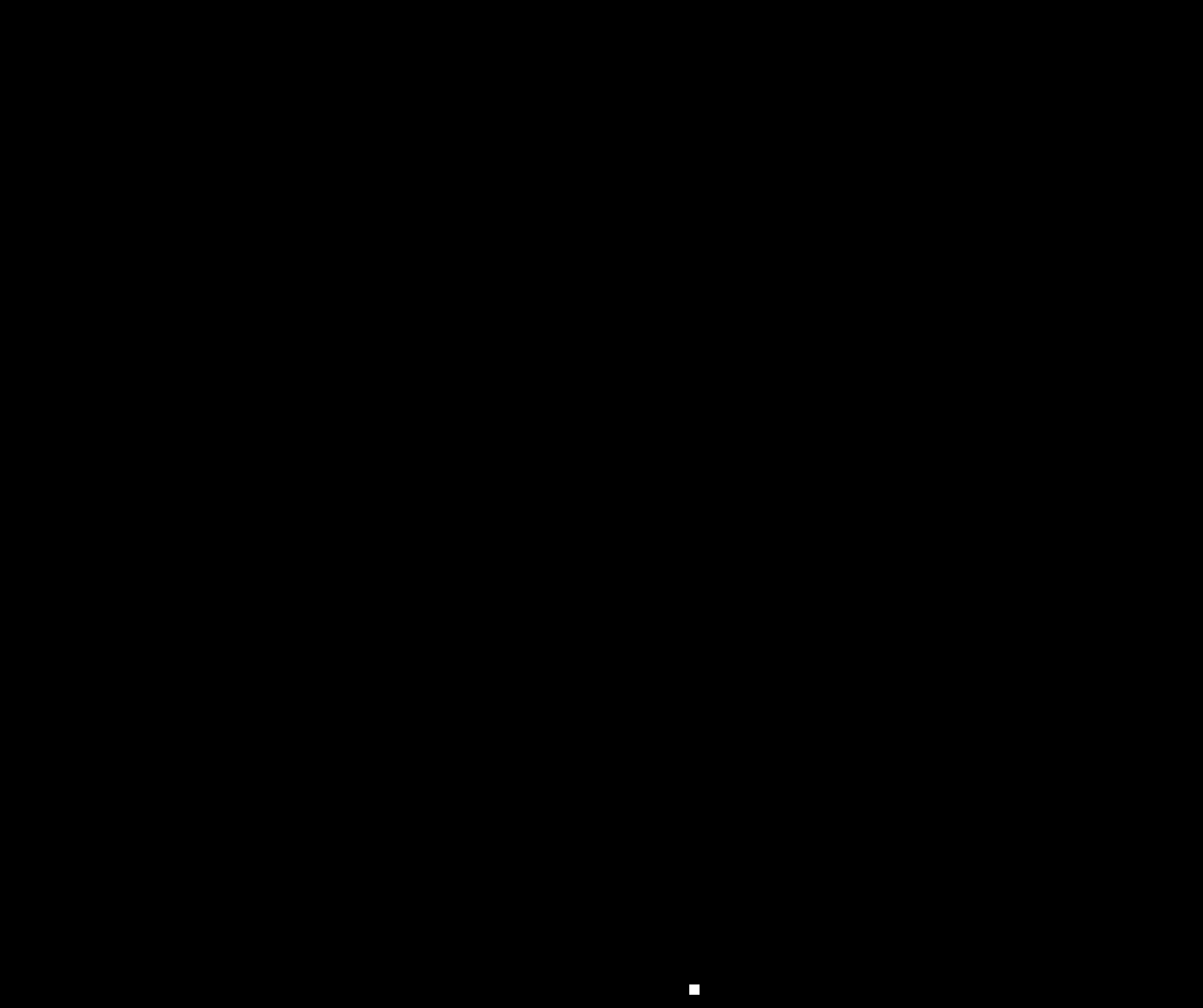

Supplement: Supplemental Information 1 — The supplemental zip file contains 3 folders: data, scripts, and license. The scripts enable denovo analysis of the data contained in the data folder, which was used to generate the figures in the manuscript. The license is GPL version2. [file peerj-06-5727-s001.zip › analysis/data/temperature/card_masks/15_mask.png]

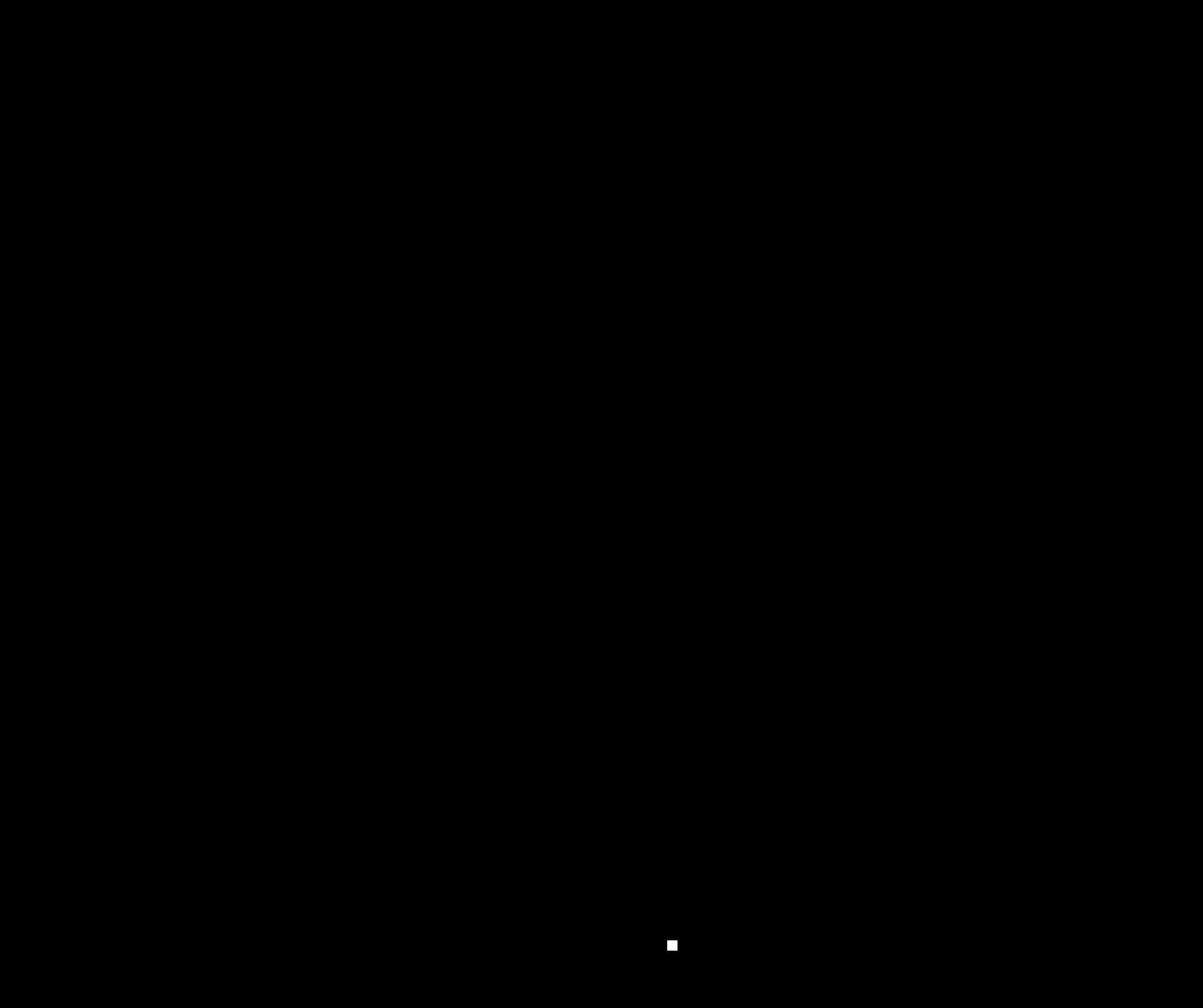

Supplement: Supplemental Information 1 — The supplemental zip file contains 3 folders: data, scripts, and license. The scripts enable denovo analysis of the data contained in the data folder, which was used to generate the figures in the manuscript. The license is GPL version2. [file peerj-06-5727-s001.zip › analysis/data/temperature/card_masks/16_mask.png]

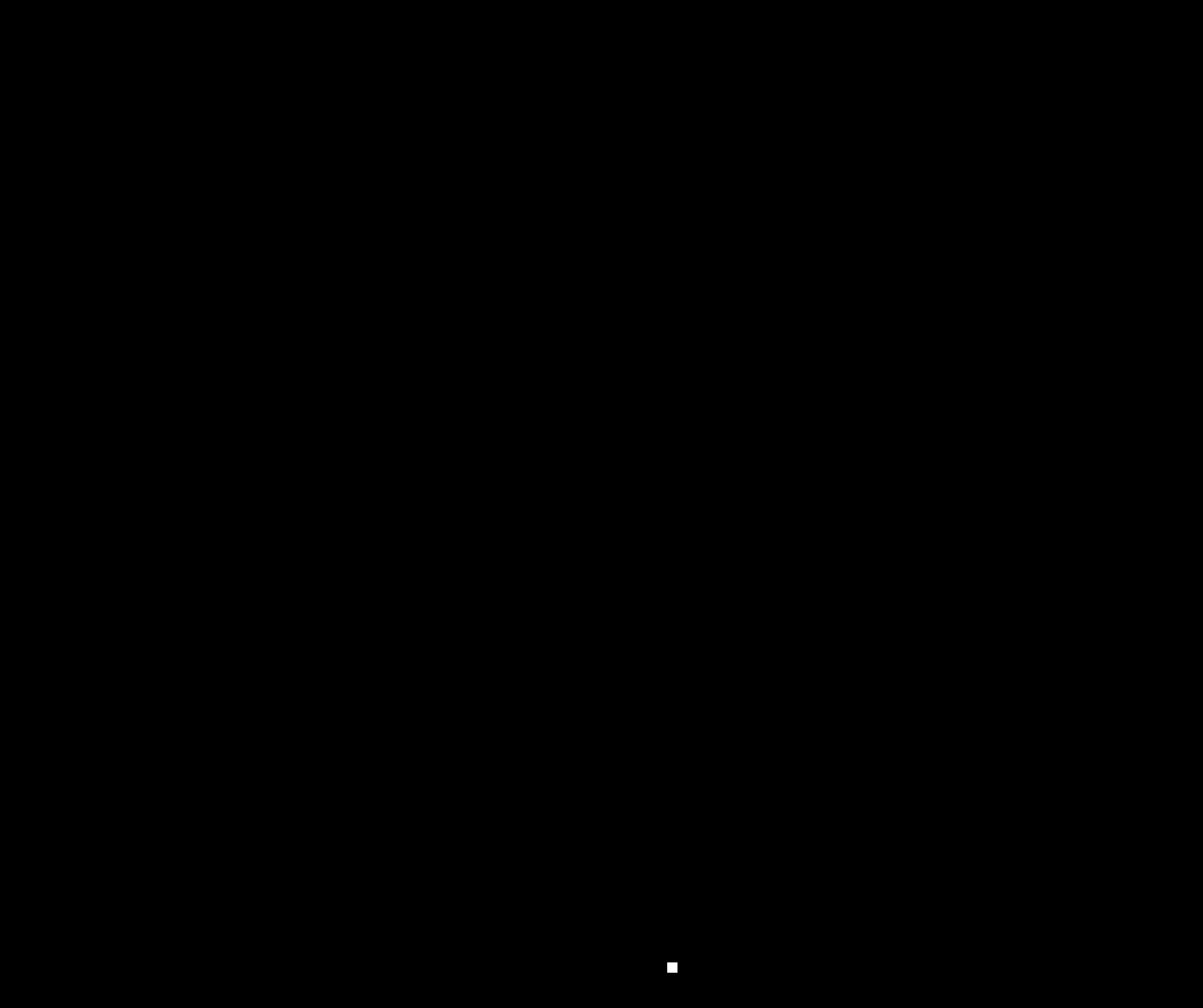

Supplement: Supplemental Information 1 — The supplemental zip file contains 3 folders: data, scripts, and license. The scripts enable denovo analysis of the data contained in the data folder, which was used to generate the figures in the manuscript. The license is GPL version2. [file peerj-06-5727-s001.zip › analysis/data/temperature/card_masks/17_mask.png]

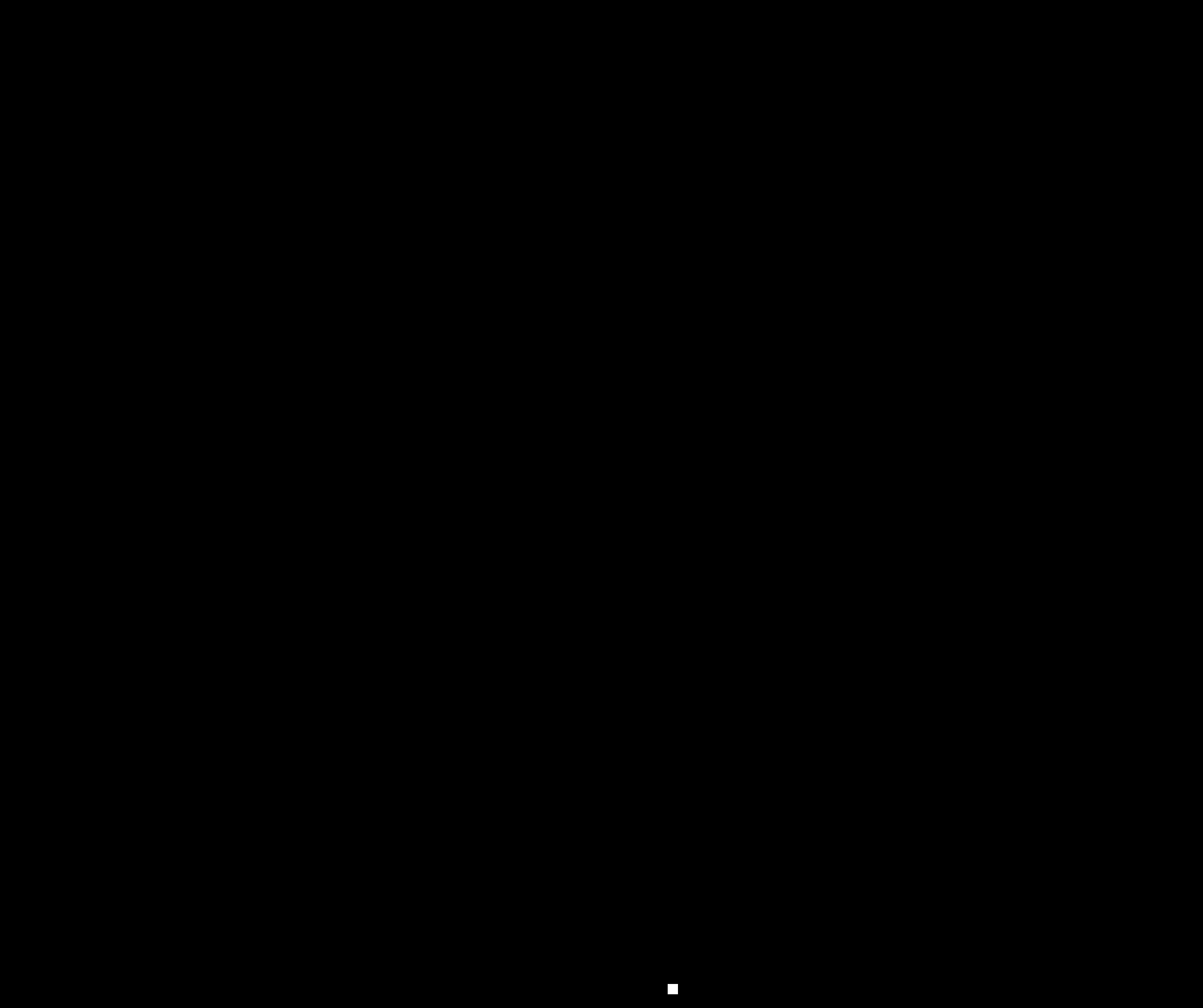

Supplement: Supplemental Information 1 — The supplemental zip file contains 3 folders: data, scripts, and license. The scripts enable denovo analysis of the data contained in the data folder, which was used to generate the figures in the manuscript. The license is GPL version2. [file peerj-06-5727-s001.zip › analysis/data/temperature/card_masks/18_mask.png]

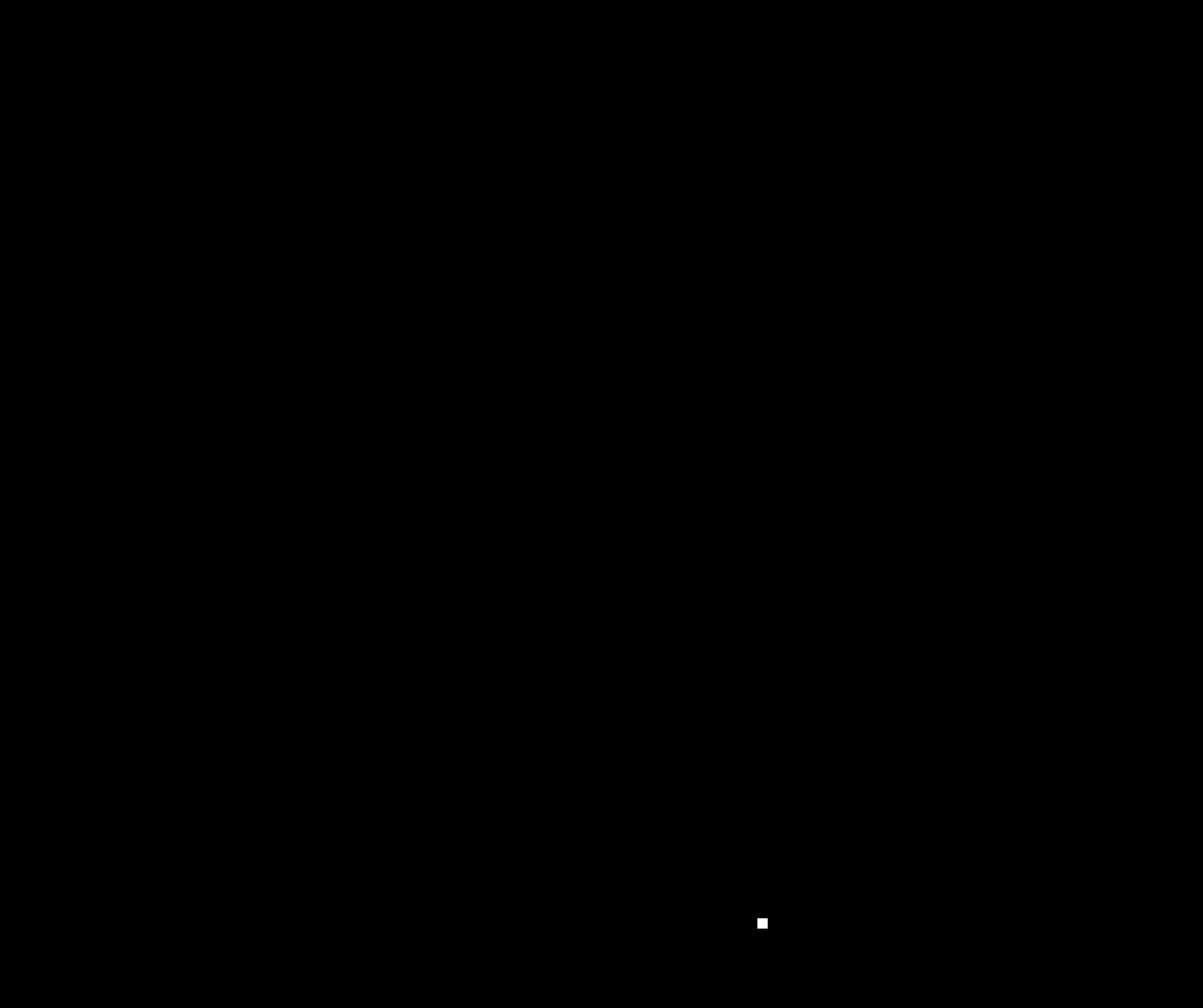

Supplement: Supplemental Information 1 — The supplemental zip file contains 3 folders: data, scripts, and license. The scripts enable denovo analysis of the data contained in the data folder, which was used to generate the figures in the manuscript. The license is GPL version2. [file peerj-06-5727-s001.zip › analysis/data/temperature/card_masks/19_mask.png]

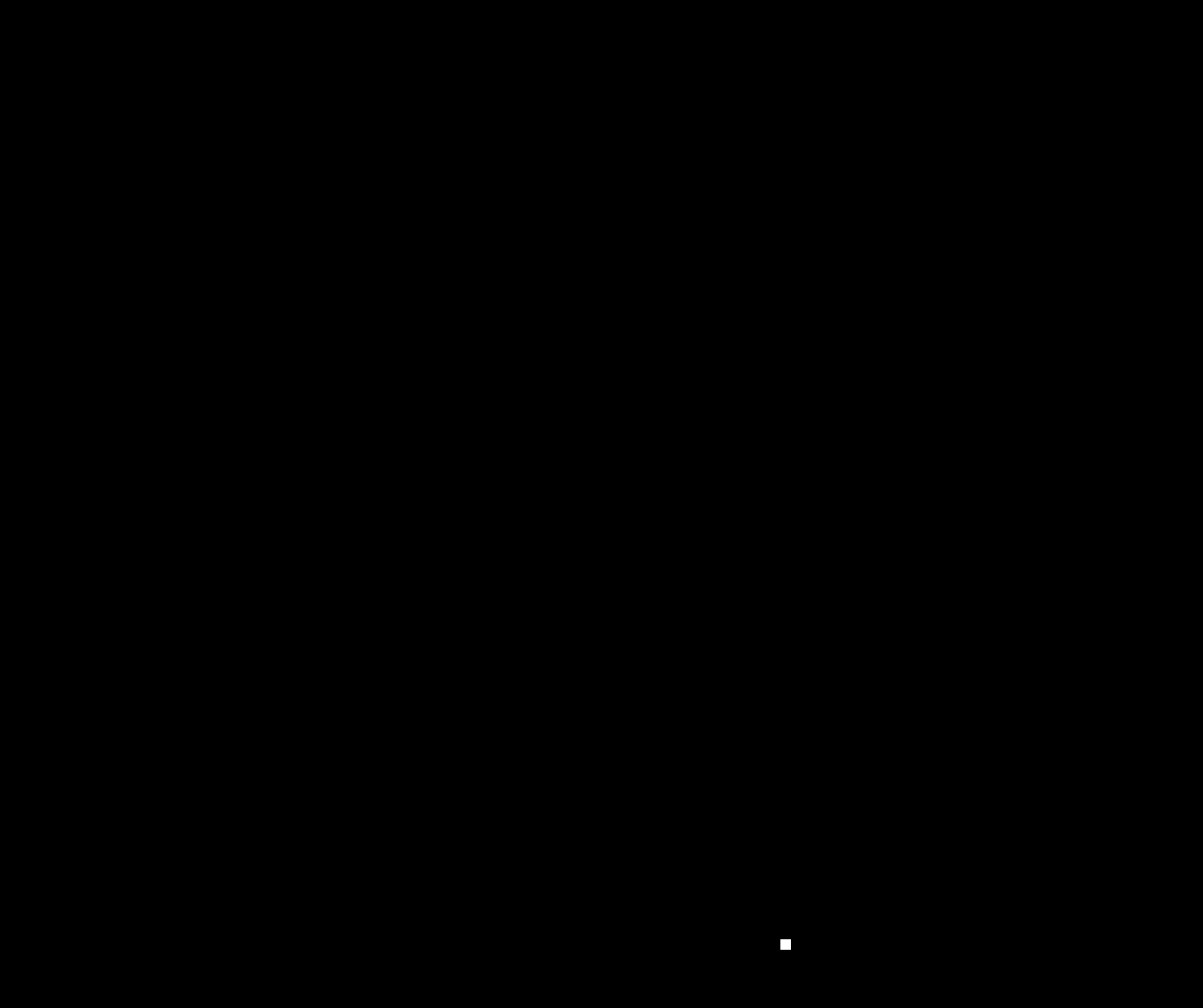

Supplement: Supplemental Information 1 — The supplemental zip file contains 3 folders: data, scripts, and license. The scripts enable denovo analysis of the data contained in the data folder, which was used to generate the figures in the manuscript. The license is GPL version2. [file peerj-06-5727-s001.zip › analysis/data/temperature/card_masks/1_mask.png]

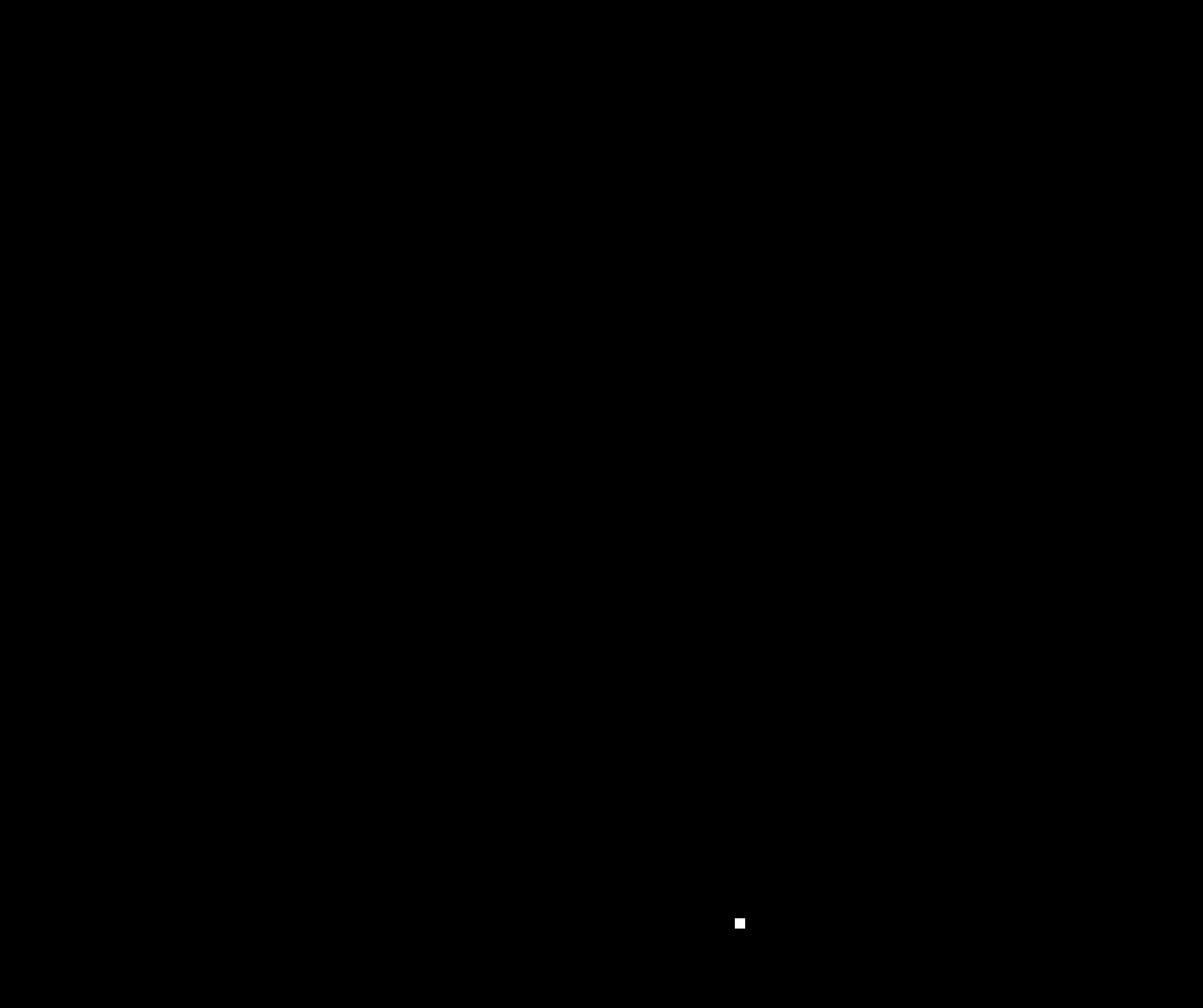

Supplement: Supplemental Information 1 — The supplemental zip file contains 3 folders: data, scripts, and license. The scripts enable denovo analysis of the data contained in the data folder, which was used to generate the figures in the manuscript. The license is GPL version2. [file peerj-06-5727-s001.zip › analysis/data/temperature/card_masks/20_mask.png]

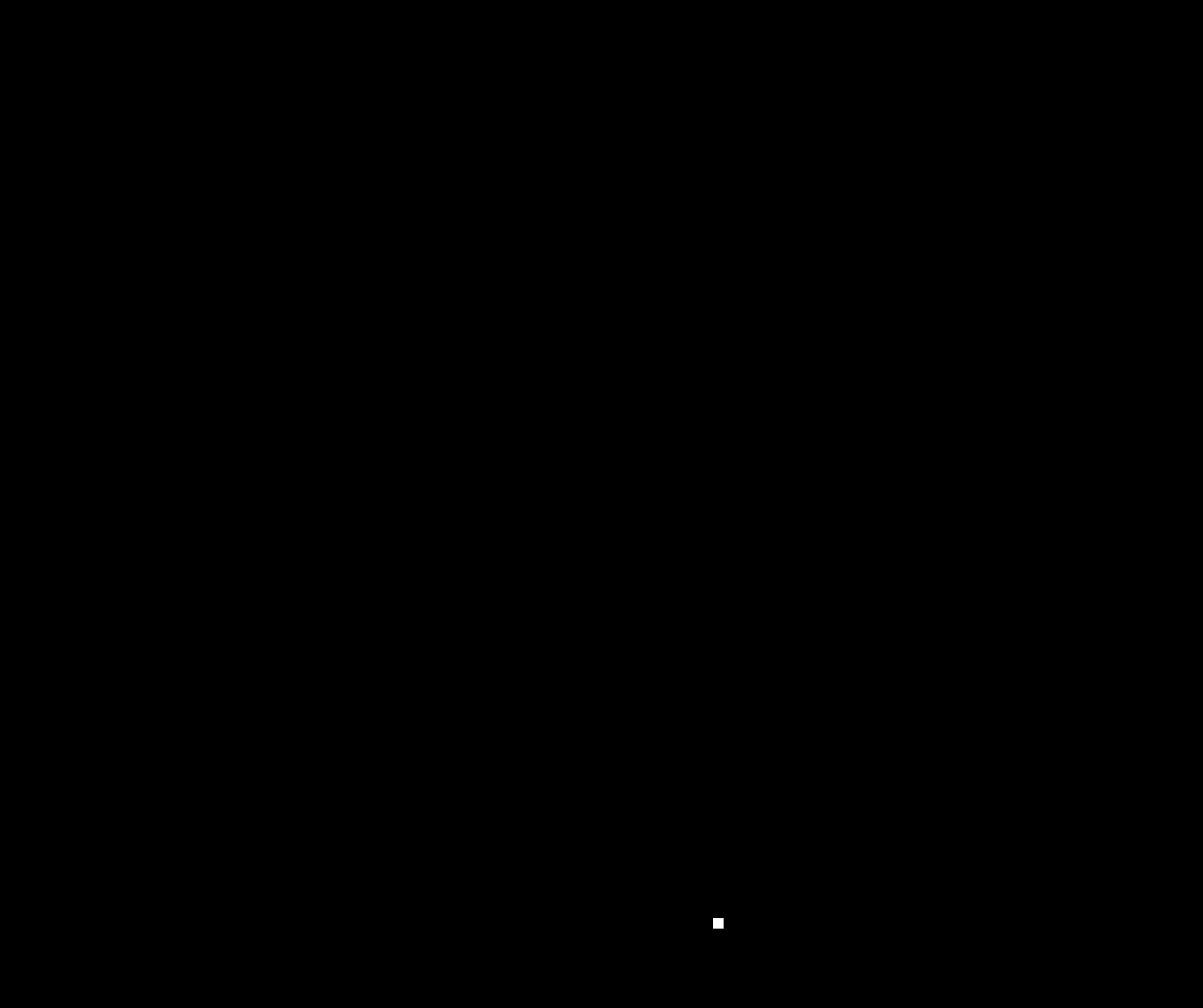

Supplement: Supplemental Information 1 — The supplemental zip file contains 3 folders: data, scripts, and license. The scripts enable denovo analysis of the data contained in the data folder, which was used to generate the figures in the manuscript. The license is GPL version2. [file peerj-06-5727-s001.zip › analysis/data/temperature/card_masks/21_mask.png]

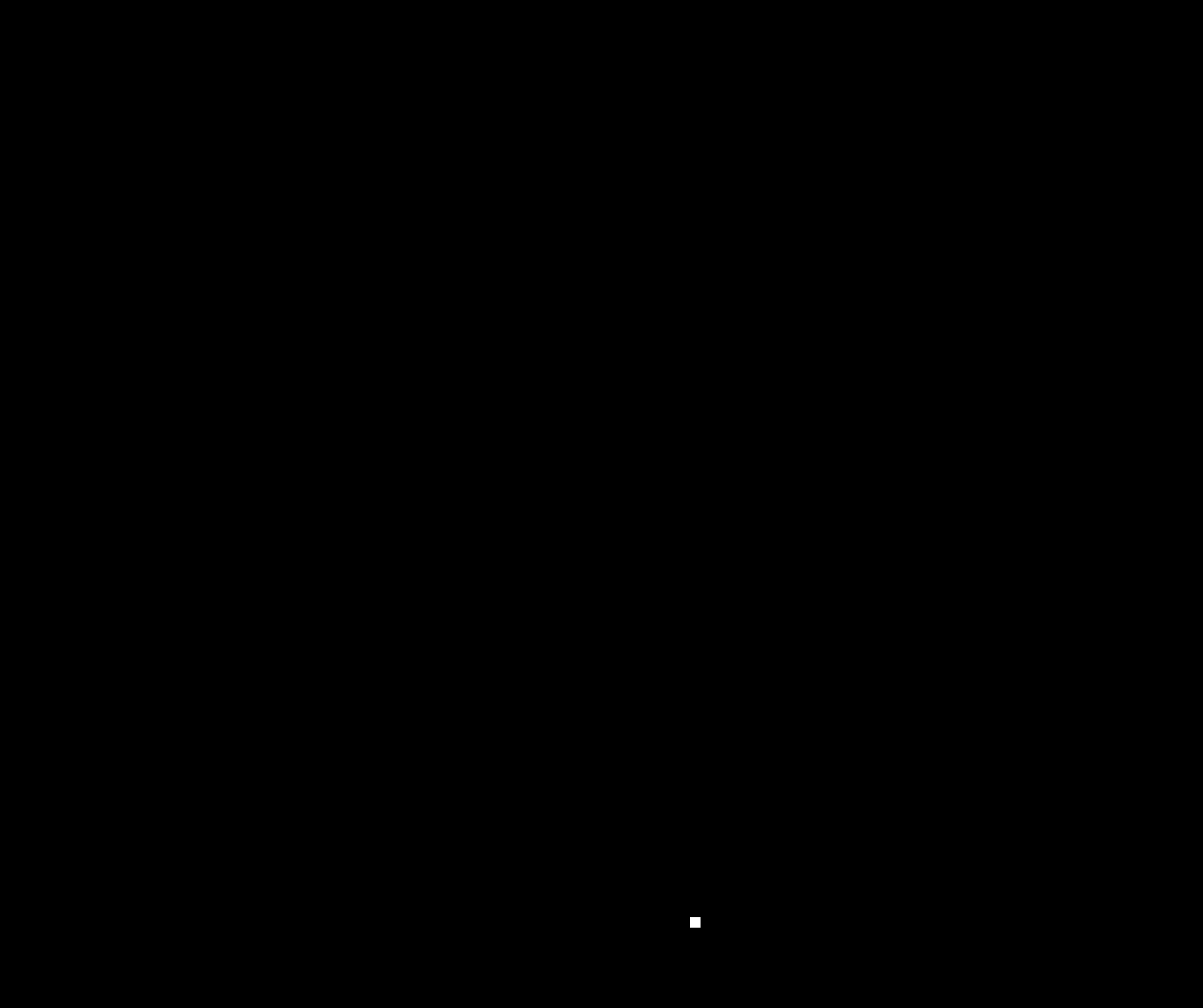

Supplement: Supplemental Information 1 — The supplemental zip file contains 3 folders: data, scripts, and license. The scripts enable denovo analysis of the data contained in the data folder, which was used to generate the figures in the manuscript. The license is GPL version2. [file peerj-06-5727-s001.zip › analysis/data/temperature/card_masks/22_mask.png]

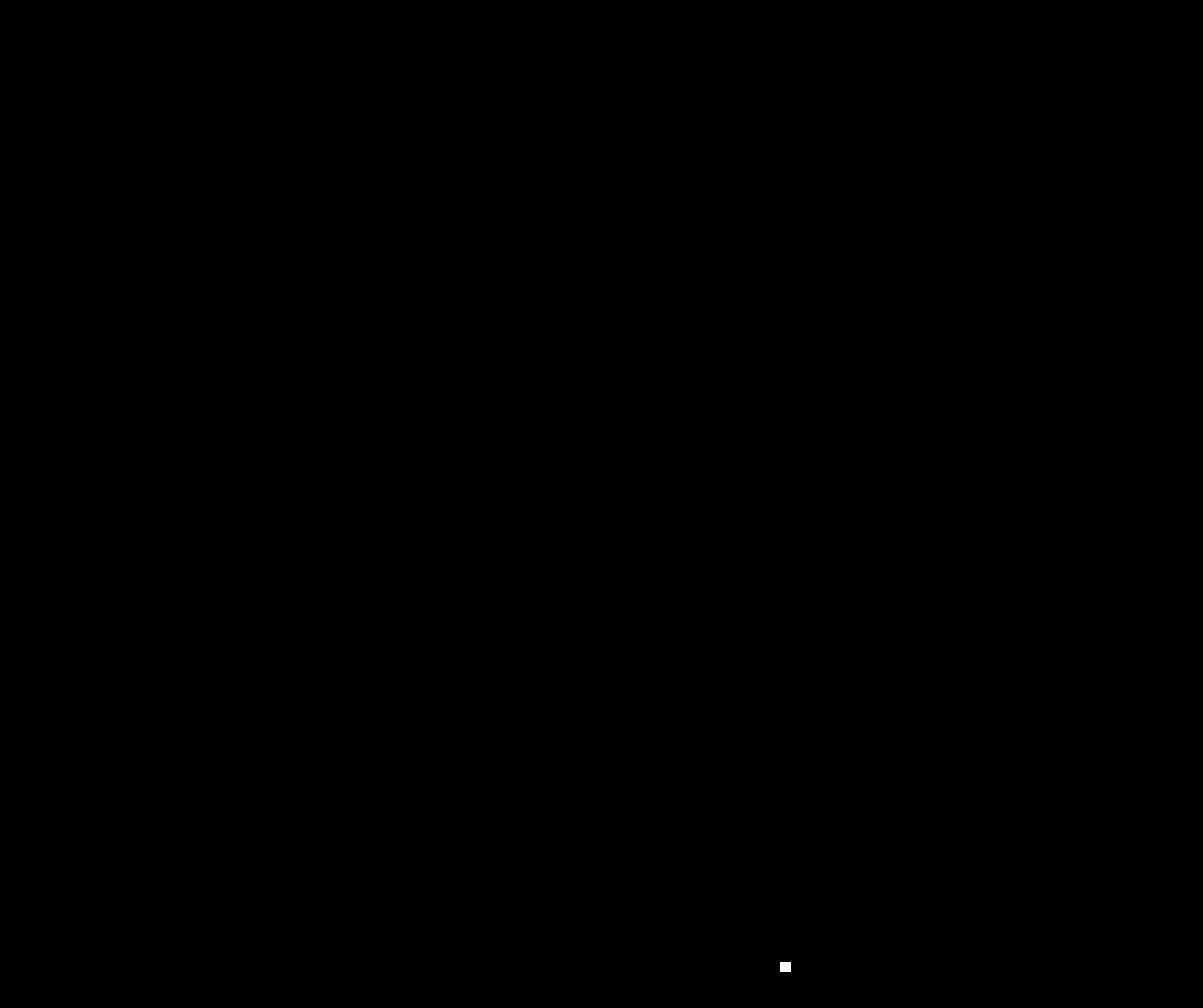

Supplement: Supplemental Information 1 — The supplemental zip file contains 3 folders: data, scripts, and license. The scripts enable denovo analysis of the data contained in the data folder, which was used to generate the figures in the manuscript. The license is GPL version2. [file peerj-06-5727-s001.zip › analysis/data/temperature/card_masks/2_mask.png]

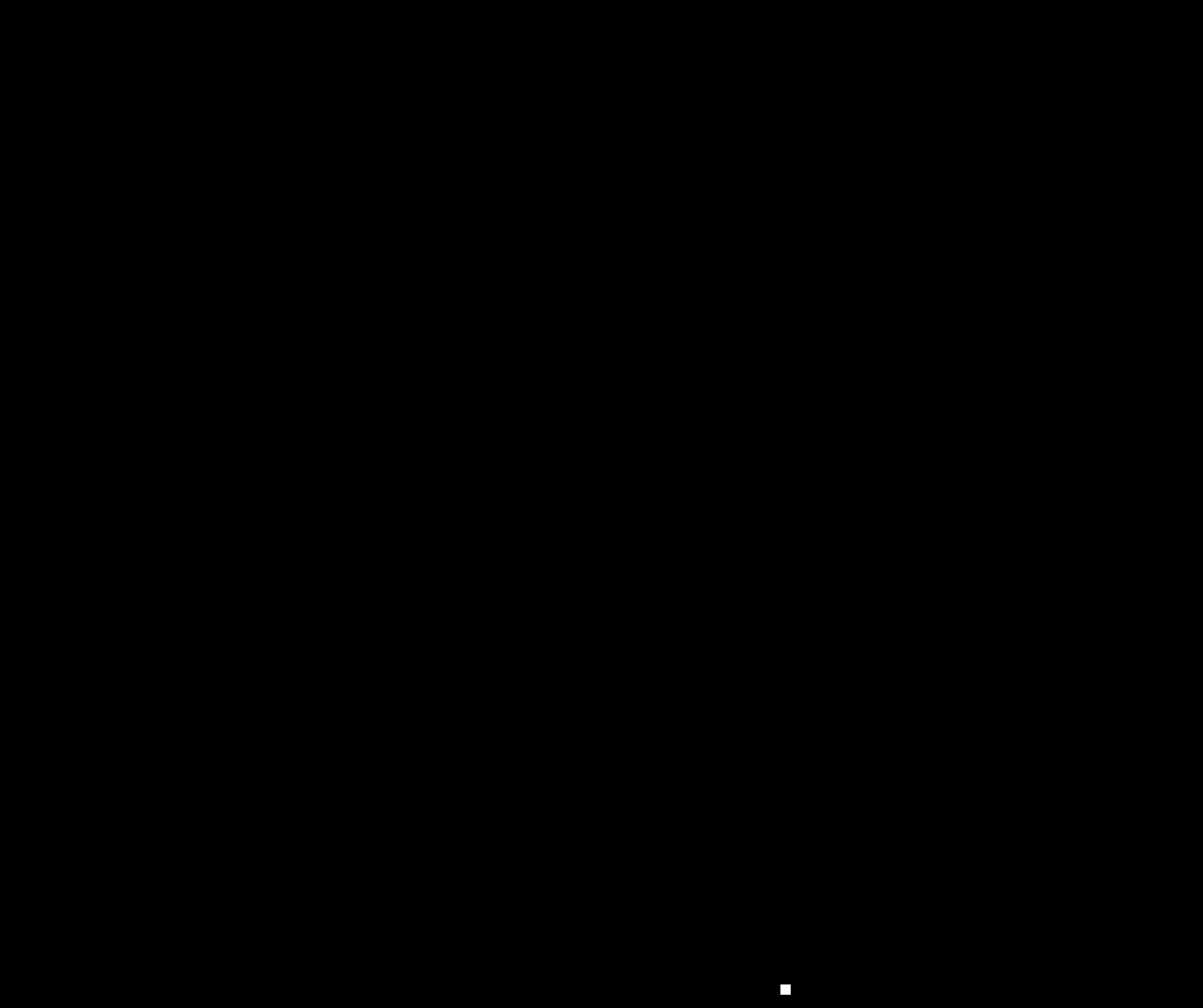

Supplement: Supplemental Information 1 — The supplemental zip file contains 3 folders: data, scripts, and license. The scripts enable denovo analysis of the data contained in the data folder, which was used to generate the figures in the manuscript. The license is GPL version2. [file peerj-06-5727-s001.zip › analysis/data/temperature/card_masks/3_mask.png]

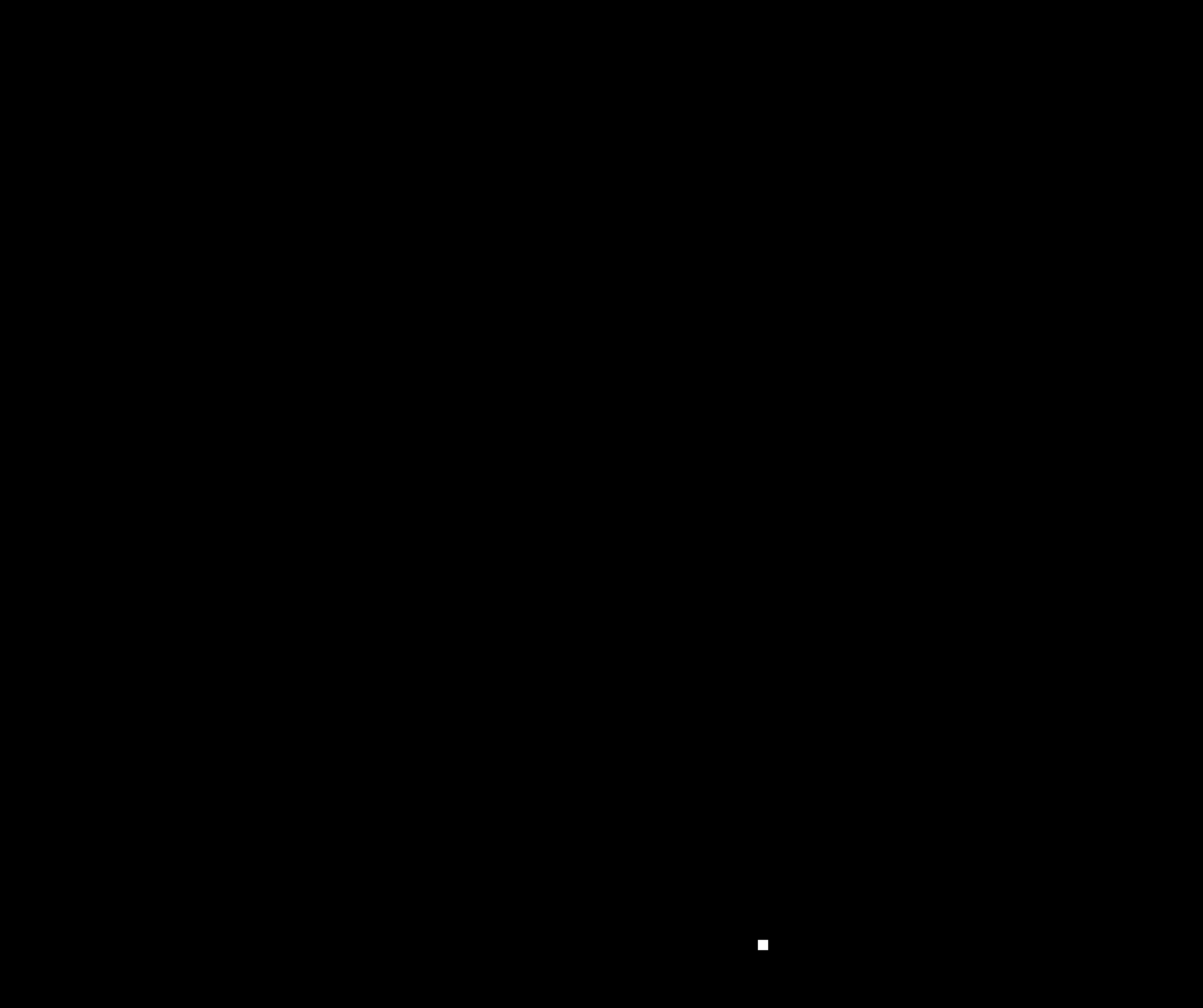

Supplement: Supplemental Information 1 — The supplemental zip file contains 3 folders: data, scripts, and license. The scripts enable denovo analysis of the data contained in the data folder, which was used to generate the figures in the manuscript. The license is GPL version2. [file peerj-06-5727-s001.zip › analysis/data/temperature/card_masks/4_mask.png]

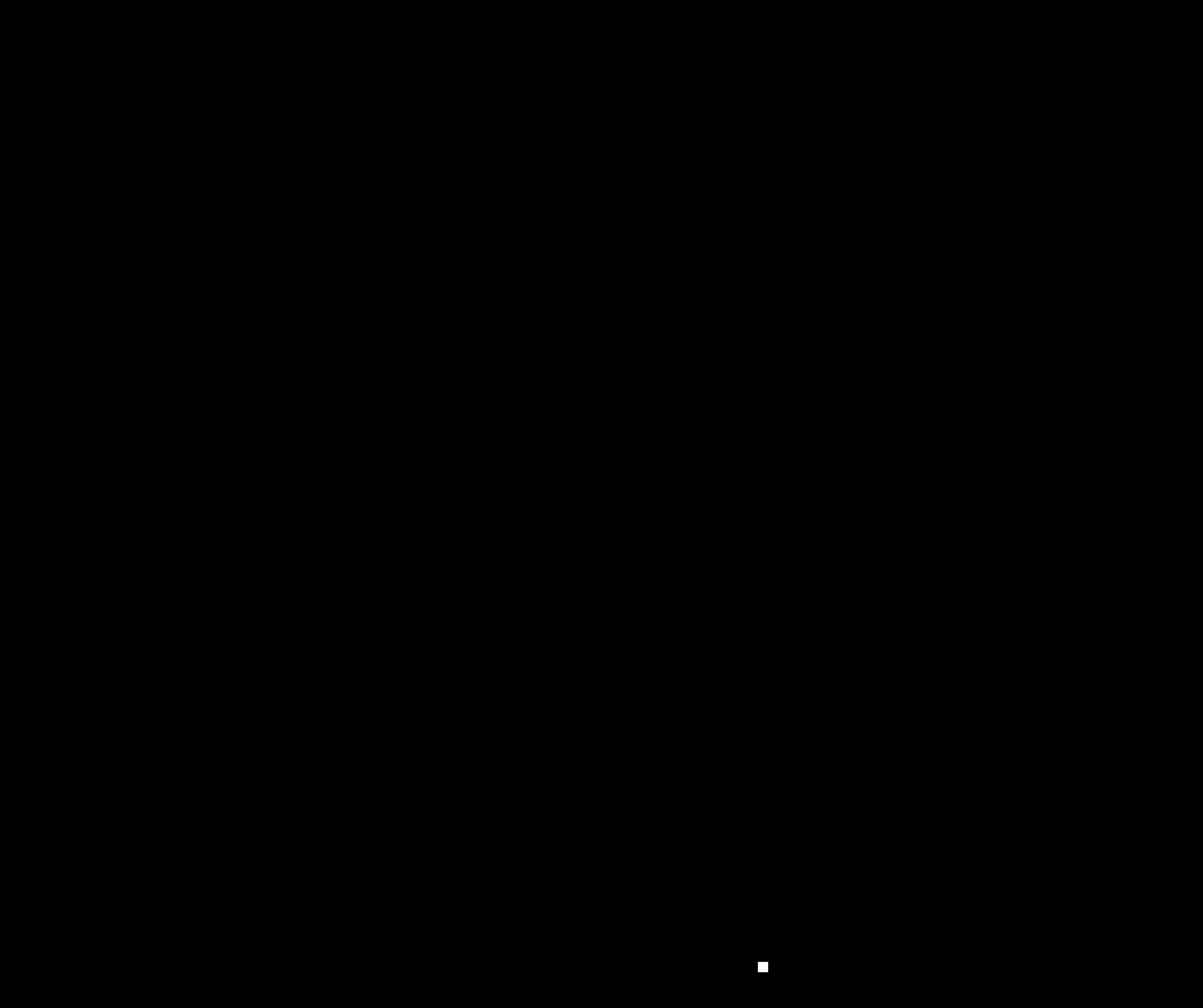

Supplement: Supplemental Information 1 — The supplemental zip file contains 3 folders: data, scripts, and license. The scripts enable denovo analysis of the data contained in the data folder, which was used to generate the figures in the manuscript. The license is GPL version2. [file peerj-06-5727-s001.zip › analysis/data/temperature/card_masks/5_mask.png]

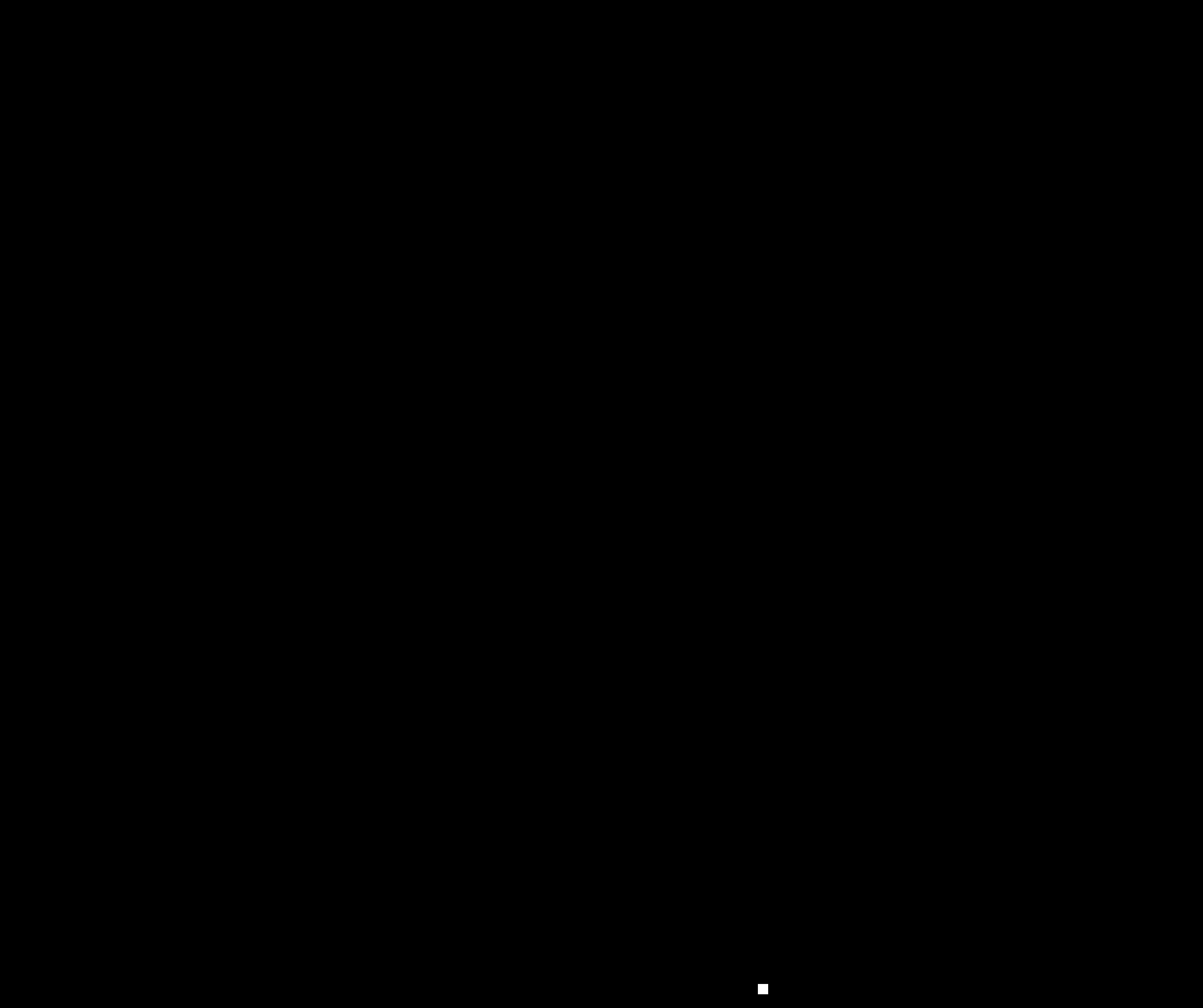

Supplement: Supplemental Information 1 — The supplemental zip file contains 3 folders: data, scripts, and license. The scripts enable denovo analysis of the data contained in the data folder, which was used to generate the figures in the manuscript. The license is GPL version2. [file peerj-06-5727-s001.zip › analysis/data/temperature/card_masks/6_mask.png]

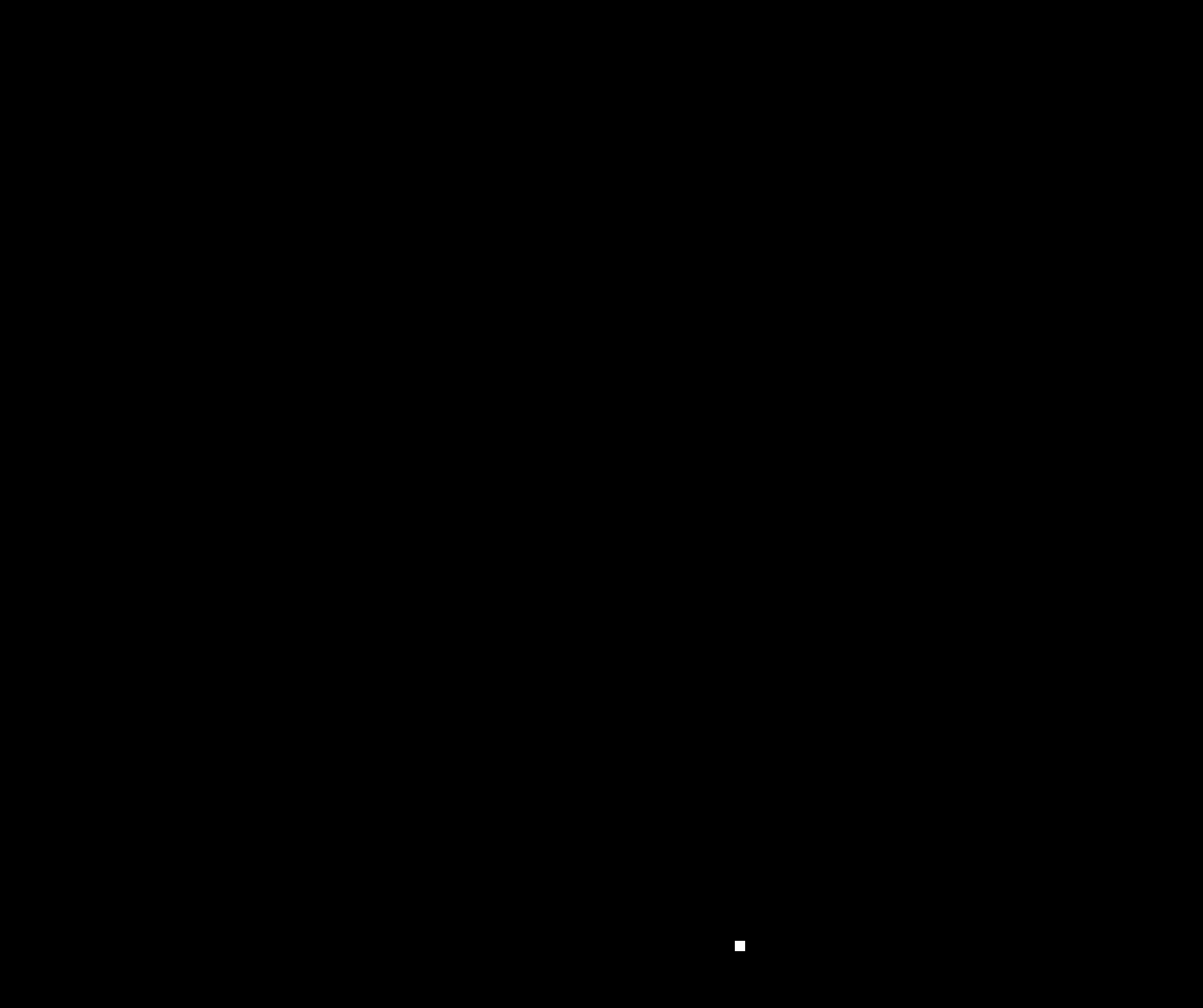

Supplement: Supplemental Information 1 — The supplemental zip file contains 3 folders: data, scripts, and license. The scripts enable denovo analysis of the data contained in the data folder, which was used to generate the figures in the manuscript. The license is GPL version2. [file peerj-06-5727-s001.zip › analysis/data/temperature/card_masks/7_mask.png]

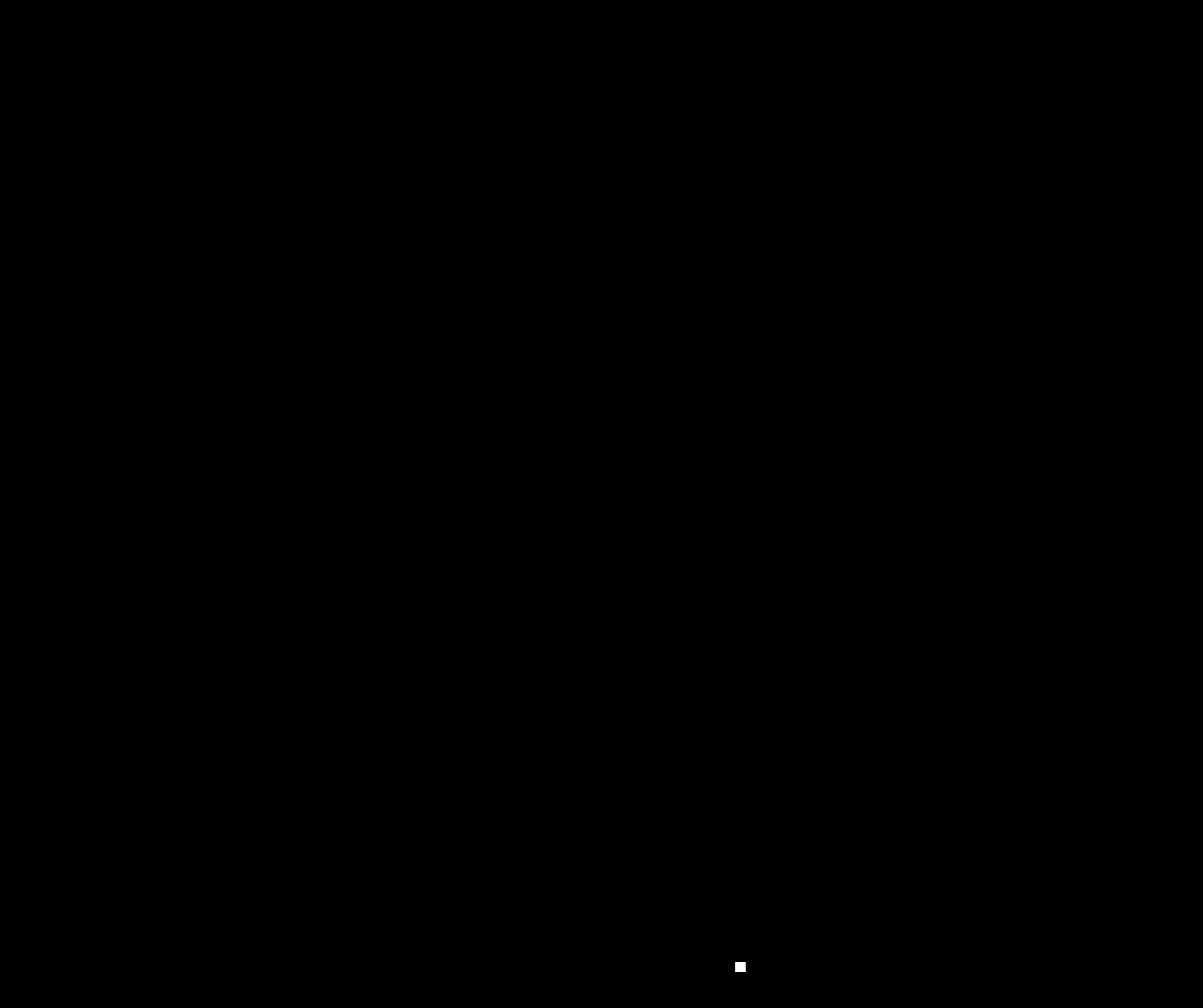

Supplement: Supplemental Information 1 — The supplemental zip file contains 3 folders: data, scripts, and license. The scripts enable denovo analysis of the data contained in the data folder, which was used to generate the figures in the manuscript. The license is GPL version2. [file peerj-06-5727-s001.zip › analysis/data/temperature/card_masks/8_mask.png]

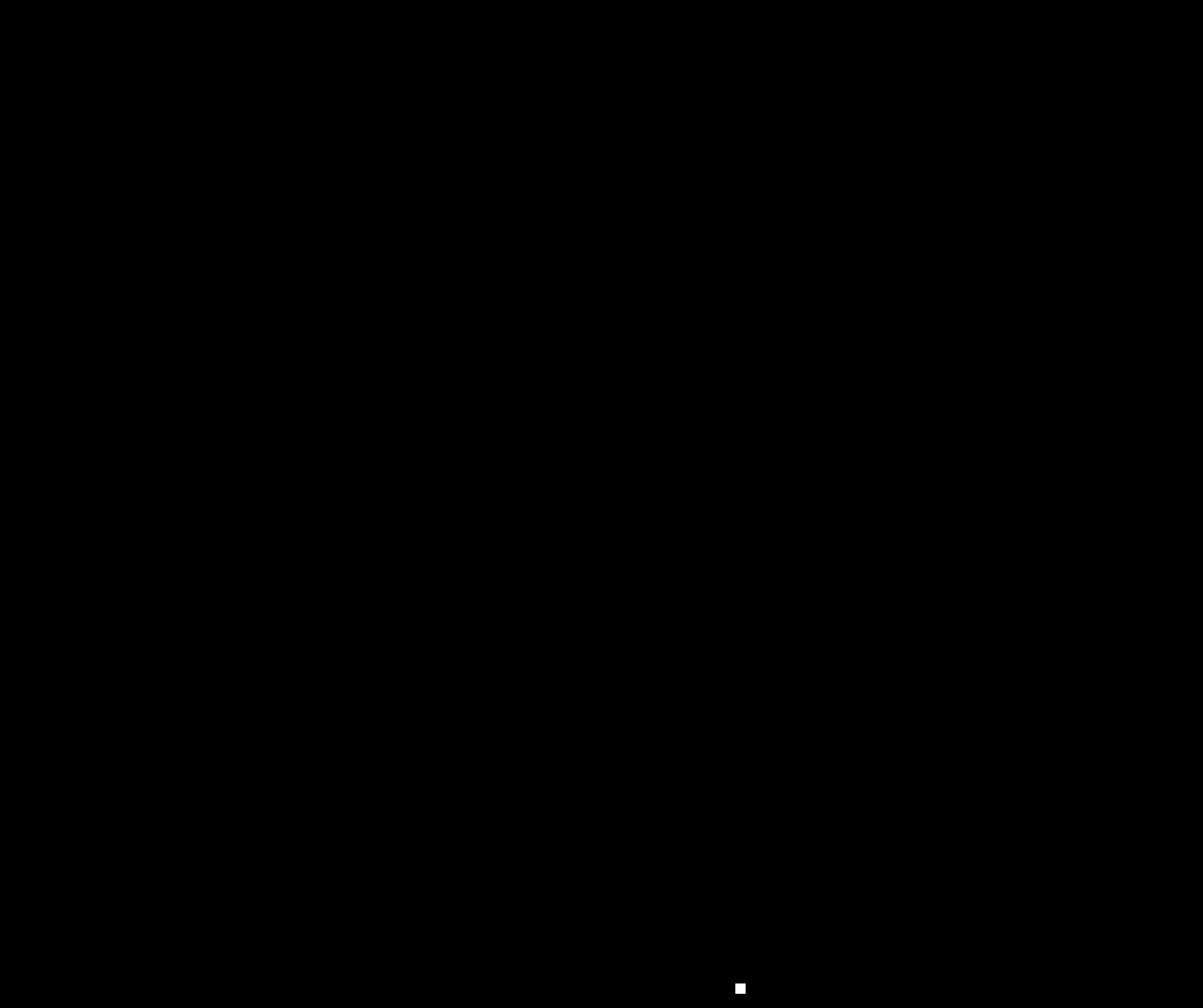

Supplement: Supplemental Information 1 — The supplemental zip file contains 3 folders: data, scripts, and license. The scripts enable denovo analysis of the data contained in the data folder, which was used to generate the figures in the manuscript. The license is GPL version2. [file peerj-06-5727-s001.zip › analysis/data/temperature/card_masks/9_mask.png]
